# Mechanisms of Mitochondrial Toxicity and Cytotoxicity Caused by *Pseudomonas aeruginosa* Pyocyanin in Human Nasal Epithelial Cells

**DOI:** 10.1002/alr.70084

**Published:** 2025-12-19

**Authors:** Joel C. Thompson, April Park, Yobouet Ines Kouakou, Zoey A. Miller, Nabil F. Darwich, Nithin D. Adappa, James N. Palmer, Ryan M. Carey, Robert J. Lee

**Affiliations:** ^1^ Department of Otorhinolaryngology Division of Rhinology University of Pennsylvania Perelman School of Medicine Philadelphia Pennsylvania USA; ^2^ Department of Physiology University of Pennsylvania Perelman School of Medicine Philadelphia Pennsylvania USA

**Keywords:** calcium, chronic rhinosinusitis, cystic fibrosis, gram‐negative bacteria, host–pathogen interactions, live cell imaging

## Abstract

**Background:**

*Pseudomonas aeruginosa* is an opportunistic pathogen in cystic fibrosis‐related chronic rhinosinusitis (CF‐CRS) that produces phenazine metabolites pyocyanin and 1‐hydroxyphenazine (1‐HP), which may have detrimental effects on mitochondria, reactive oxygen species (ROS), Ca^2+^ signaling, and apoptosis. However, prior studies utilized lung cancer cells or dissociated animal cells. We sought to better define human nasal epithelial responses to phenazines, including the role of Ca^2+^.

**Methods:**

Live cell imaging was used to measure Ca^2+^ and mitochondrial function in RPMI2650 nasal carcinoma cells and primary human nasal epithelial cells (HNECs) cultured in submersion and at air–liquid interface (ALI). Gene expression was measured by quantitative PCR. Ciliary beat frequency (CBF) was quantified by high‐speed imaging.

**Results:**

Pyocyanin, but not 1‐HP, increased mitochondrial Ca^2+^ dependent on phospholipase C and endoplasmic reticulum (ER) Ca^2+^ release, correlating with protein kinase C activation. Mitochondrial membrane potential decreased and mitochondrial ROS increased with both pyocyanin and 1‐HP in a Ca^2+^‐independent manner. Both pyocyanin and 1‐HP decreased viability of RPMI2650s and other squamous carcinoma cell lines over 24 h, whereas HNECs survived, possibly due to differential regulation of protein homeostasis genes, including activating transcription factor 6 (*ATF6*). Mitochondrial ROS was enhanced in CF‐CRS ALIs, which may explain why pyocyanin reduced CBF in CF but not non‐CF ALIs.

**Conclusions:**

Ca^2+^ signaling is not required for phenazine mitochondrial toxicity. The greater sensitivity of cancer cells to phenazine cytotoxicity necessitates use of primary cells when studying host responses to bacterial phenazines. Enhanced ROS production and ciliotoxicity in CF‐CRS may contribute to susceptibility to *P. aeruginosa* infection.

## 1 Introduction

*Pseudomonas aeruginosa* is an opportunistic pathogen that primarily infects immunocompromised individuals such as burn victims or cancer patients and causes fatal respiratory infections in patients with cystic fibrosis (CF) [1, 2]. The prevalence of multidrug‐resistant *P. aeruginosa* is increasing, making it a significant public health threat in these patient populations [3]. Understanding the interactions of *P. aeruginosa* with host cells may aid the development of new therapies that might circumvent antibiotic resistance [4]. We previously studied activation of bitter taste receptors (taste family 2 receptors or T2Rs) by *P. aeruginosa* acyl‐homoserine lactone (AHLs) and quinolone quorum sensing molecules [5]. One *P. aeruginosa* AHL, 3‐oxo‐C12HSL, induces short‐term innate defense responses in nasal epithelial cells through activation of T2R38 [5] but can also induce longer‐term apoptosis responses in oral and nasal epithelial cells through activation of T2R14 [6]. T2R14 activation causes mitochondrial Ca^2+^ (Ca^2+^_mito_) overload and excessive mitochondrial reactive oxygen species (mtROS) production that inhibits the ubiquitin‐proteasome pathway [7]. We hypothesize that other *P. aeruginosa* metabolites interact with T2Rs or other host G protein‐coupled receptors (GPCRs) that may be potentially druggable therapeutic targets.

*P. aeruginosa* synthesizes several other cytotoxic virulence factors [8], including phenazine metabolites pyocyanin and 1‐hydroxyphenazine (1‐HP), before and during microcolony and biofilm development [1, 9]. Pyocyanin functions in virulence and quorum sensing [1], with concentrations in late‐stationary cultures or biofilms ≥100–300 µM [10] and concentrations >100 µM reported in CF patient sputum [11] and infected human ear secretions [12]. Pyocyanin is a “redox‐active” compound suggested to induce apoptosis through superoxide production and mitochondrial dysfunction [1, 2, 13], but the exact mechanisms involved are unclear. In human monocyte‐derived macrophages, pyocyanin did not induce apoptotic caspase‐3 or caspase‐7 activation after 24 h [14], whereas pyocyanin did activate caspase‐3 and apoptosis in MCF‐7 breast cancer cells [15], hinting at differential targets of pyocyanin between cancer and non‐cancer cells. Other studies have shown that pyocyanin increases activating transcription factor 6 (ATF6) in non‐cancerous NRK‐52E cells [16]. ATF6 plays a role in the endoplasmic reticulum (ER) unfolded protein response (UPR^ER^), though how it fits into pyocyanin toxicity has not yet been demonstrated. Pyocyanin has been suggested to have potential anti‐cancerous effects via apoptosis as well as potential antifungal or antibacterial properties [1, 2].

Many of the pathways above are regulated by Ca^2+^ signaling, which could be therapeutically targeted to manipulate the effects of phenazines, for example, to reduce detrimental effects during infection or to enhance cytotoxic effects as part of an anticancer therapy. It is unknown if pyocyanin or 1‐HP activates Ca^2+^ responses in primary nasal epithelial cells, and if so, if this is GPCR‐related. Ca^2+^ is a master regulator of many cellular processes and has a biphasic relationship with mitochondrial function. Although Ca^2+^ is required for proper mitochondrial function, too much mitochondrial Ca^2+^ leads to cell death [17, 18]. Another goal here was to test if mitochondrial dysfunction and epithelial damage are linked to excessive mitochondrial Ca^2+^ influx, as observed with 3‐oxo‐C12HSL [6]. Only one study of A549 lung cancer cells linked pyocyanin to increased cytosolic Ca^2+^ via ER Ca^2+^ release [19], which would fit with activation of a GPCR. No studies have examined 1‐HP in Ca^2+^ signaling. Other studies have shown that pyocyanin and 1‐HP can diminish or even completely inhibit ciliary beating in airway epithelial cells [20, 21, 22], as we observed with activation of T2R1 in nasal epithelial cells [23]. However, these studies were largely done in isolated dissociated cells, which may not reflect what happens in an intact epithelium. We also sought to determine if phenazines activated T2Rs and shared similar mechanisms of apoptosis induction with 3‐oxo‐C12HSL. As we previously showed that T2R responses are altered in primary nasal epithelial cells from people with CF [24], a secondary goal here was to determine if cells from CF individuals had altered responses to pyocyanin or 1‐HP. Defining the effects of these *P. aeruginosa* metabolites on nasal epithelial cells will reveal insights into host–pathogen interactions specifically relevant to cystic fibrosis‐related chronic rhinosinusitis (CF‐CRS).

## 2 Materials and Methods

RPMI2650 (ATCC CCL‐30) and NCI‐H520 (ATCC HTB‐182) cells were from ATCC (Manassas, VA, USA), whereas UM‐SCC‐47 (SCC‐47; Millipore SCC071) was from MilliporeSigma (St. Louis, MO, USA), and all were cultured as described [25]. Cells were cultured in Minimal Essential Medium with Earle's salts and l‐glutamine (Corning; Glendale, AZ, USA) with 10% (v/v) fetal bovine serum (MedSupply Partners; Atlanta, GA, USA), 1% (v/v) penicillin/streptomycin mix (Gibco; Gaithersburg, MD, USA), and 1% (v/v) MEM nonessential amino acids (Gibco). Cell media was changed twice per week, and cells were passaged between 70% and 80% confluence.

Human nasal epithelial cells (HNEC) were extracted via cytology brushings, as previously described [23], from patients undergoing sinonasal surgery for CRS or other procedures (e.g., trans‐nasal approaches to the skull base) with institutional review board (IRB) approval (protocol #800614) and with written informed consent. All work was done in accordance with the University of Pennsylvania guidelines for use of residual clinical material, the US Department of Health and Human Services code of federal regulation Title 45 CFR 46.116, and the Declaration of Helsinki. Inclusion criteria were patients ≥18 years of age undergoing sinonasal surgery. Exclusion criteria included use of antibiotics, oral corticosteroids, or biologics within 1 month of surgery. Patients with a history of systemic inheritable disease (e.g., granulomatosis with polyangiitis, systemic immunodeficiencies), not including CF, were excluded, as were individuals ≤18 years of age, pregnant women, and cognitively impaired persons. Full details of experimental protocols and statistical methods are provided in the Supporting Information section.

## 3 Results

### 3.1 Pyocyanin, but Not Related 1‐HP, Increases Mitochondrial Ca^2+^ Through an Unknown Receptor That Signals Through Phospholipase C (PLC)

**FIGURE 1:**
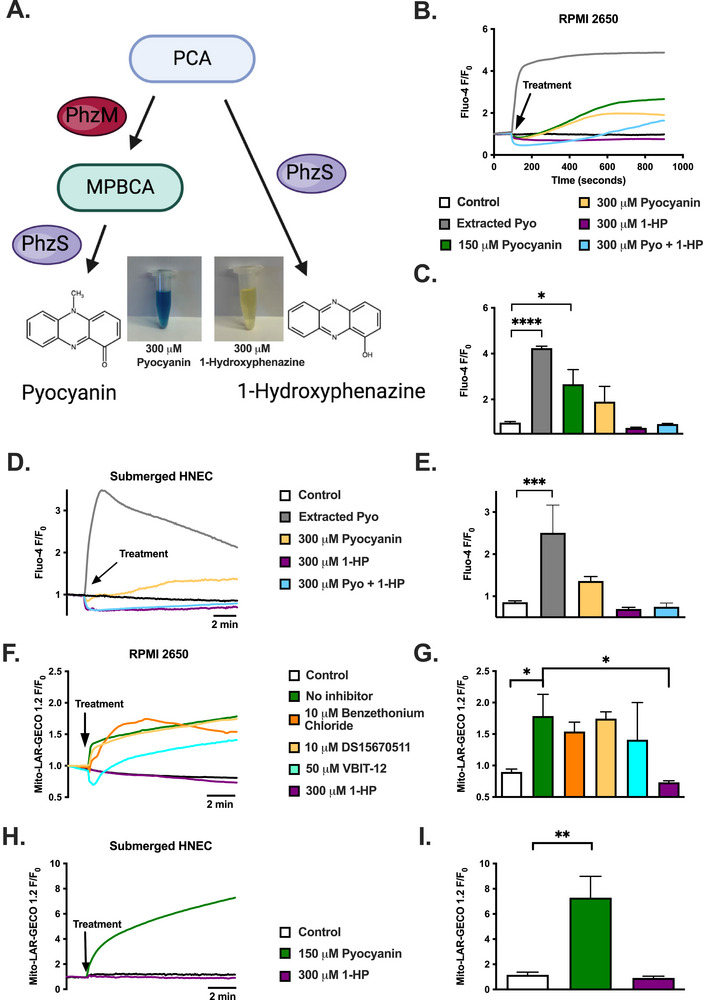
Pyocyanin induces cytoplasmic and mitochondrial Ca^2+^ responses in RPMI2650 and submerged HNEC, whereas 1‐hydroxyphenazine does not. (A) Phenazine‐1‐carboxylic acid (PCA) precursor is converted to 5‐methylphenazine‐1‐carboxylic acid betaine (MPBCA) by the methyltransferase PhzM [71], which is converted to pyocyanin by hydroxylase PhzS [72]. In the absence of PhzM or with buildup in PCA, PhzS also directly converts PCA to 1‐hydroxyphenazine [73]. (B) Average traces (*n* ≥ 3 independent experiments) of Fluo‐4 intracellular Ca^2+^ (Ca^2+^_i_) responses (fluorescence/fluorescence at time 0; *F/F*_0_) in RPMI2650s induced by extracted pyocyanin (Pyo) from ATCC 27853 (measured at 18.2 µM), or 150, or 300 µM Pyo, or 300 µM Pyo + 1‐hydroxyphenazine (1‐HP) but not 300 µM 1‐HP. (C) Ca^2+^_i_ (mean ± SEM) 13 min posttreatment in RPMI2650s. (D) Average traces (*n* ≥ 3 independent experiments) of Fluo‐4 Ca^2+^_i_ responses in submerged primary human nasal epithelial cells (HNECs) induced by extracted Pyo from ATCC 27853 (33.9 µM), or 300 µM Pyo but not 300 µM 1‐HP or 300 µM Pyo + 1‐HP. (E) Ca^2+^_i_ (mean ± SEM) 13 min posttreatment in submerged HNECs. (F) Average traces (*n* = 3 independent experiments) of Mito‐LAR‐GECO1.2 mitochondrial Ca^2+^ (Ca^2+^_mito_) responses in RPMI2650s induced by 150 µM Pyo but not 300 µM 1‐HP. This was not significantly different with mitochondrial Ca^2+^ uniporter (MCU) inhibitors (10 µM benzethonium chloride or DS16570511) or with voltage‐dependent anion channel (VDAC1) inhibitor (50 µM VBIT‐12). All pre‐treated cells were subsequently treated with 150 µM pyocyanin. HBSS (vehicle control) and 1‐HP‐treated wells were not pre‐treated with any inhibitors. (G) Ca^2+^_mito_ (mean ± SEM) 10 min posttreatment in RPMI2650s. (H) Average Mito‐LAR‐GECO1.2 traces of Ca^2+^_mito_ responses in submerged HNECs induced by 150 µM pyocyanin only (*n* ≥ 3 independent experiments using cells from three patients per condition). (I) Ca^2+^_mito_ (mean ± SEM) 13 min posttreatment in submerged HNEC. Significance in bar graphs determined by one‐way ANOVA with Dunnett's posttest; *p < 0.05, **p < 0.01, ***p < 0.001.

Pyocyanin and 1‐HP are produced from the same precursor, phenazine‐1‐carboxylic acid, with the ratio of pyocyanin to 1‐hydroxyphenzine dictated by the balance of PhzM and PhzS enzymatic activity (Figure 1A). Both pyocyanin and 1‐HP are secreted by lab *P. aeruginosa* strains during stationary phase [26]. We first investigated the ability of phenazines to increase intracellular Ca^2+^ (Ca^2+^_i_) by imaging submerged primary HNECs and RPMI2650 nasal carcinoma cells loaded with Ca^2+^ indicator dye Fluo‐4. Interestingly, pyocyanin induced slow‐onset but sustained Ca^2+^_i_ responses in both cell types, whereas 1‐HP did not (Figure 1B–E), appearing in some cases to lower Ca^2+^_i_ (Figure 1B,D). Interestingly, the combination of pyocyanin and 1‐HP negated the Ca^2+^_i_ response with pyocyanin alone (Figure 1C,E). When we tested pyocyanin extracted as previously described [27, 28] from cultures of a pyocyanin‐producing strain of *P. aeruginosa*, we also observed robust Ca^2+^_i_ responses with Fluo‐4 in both RPMI2650s and submerged primary HNECs (Figure 1D,E).

Mitochondria are important Ca^2+^ buffering organelles that can take up Ca^2+^ when Ca^2+^_i_ is elevated [18] or directly take up ER Ca^2+^ from mitochondrial‐ER contact points [29]. Pyocyanin, but not 1‐HP, induced elevations of mitochondrial Ca^2+^ (Ca^2+^_mito_; Figure 1F–I). However, the Ca^2+^_mito_ increase with pyocyanin was not significantly decreased by inhibitors of mitochondrial outer membrane Ca^2+^ channels (voltage‐dependent anion channel [VDAC1] inhibitor VBIT‐12 [30]) or inner membrane Ca^2+^ channels (mitochondrial Ca^2+^ uniporter [MCU] inhibitors benzethonium chloride [31] or DS15670511 [32]) in RPMI2650s (Figure 1F–G), suggesting alternative noncanonical Ca^2+^ influx pathways are involved. This contrasts with our observations of 3‐oxo‐C12HSL‐induced Ca^2+^_mito_ signaling, which is blocked by both MCU inhibitors. These data suggest that pyocyanin, unlike 3‐oxo‐C12HSL, increases Ca^2+^_mito_ through a non‐MCU pathway.

**FIGURE 2:**
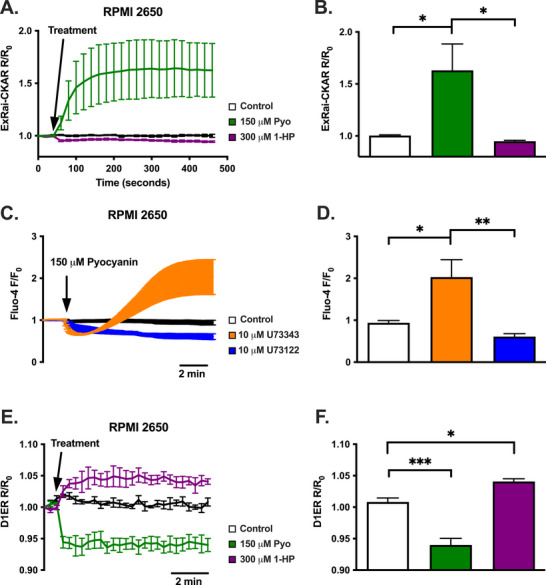
Pyocyanin induces PKC activation and ER calcium release, whereas 1‐hydroxyphenazine does not, and pyocyanin‐induced Ca^2+^ is diminished by PLC inhibition. (A) Average ExRai‐CKAR traces (mean ± SEM; *n* = 6 independent experiments; fluorescence ratio/fluorescence ratio at time 0; *R/R*_0_) of PKC activity in RPMI2650s. Responses were induced by 150 µM pyocyanin (Pyo) but not 300 µM 1‐hydroxyphenazine (1‐HP). (B) PKC activity (mean ± SEM) 6 min posttreatment in RPMI2650s. (C) Average traces (mean ± SEM; *n* = 3 independent experiments) of Fluo‐4 Ca^2+^_i_ responses (fluorescence/fluorescence at time 0; *F/F*_0_) in RPMI2650 that were pre‐treated 1 h before with U73122 PLC inhibitor versus U73343 inactive control. A solution of 150 µM pyocyanin Ca^2+^_i_ responses were blocked by U73122. (D) Ca^2+^_i_ (mean ± SEM) 10 min posttreatment in RPMI2650. (E) Average traces (mean ± SEM; *n* = 3 independent experiments; fluorescence ratio/fluorescence ratio at time 0; *R/R*_0_) of ER Ca^2+^ in RPMI2650s transfected with D1ER. Decreases in ER Ca^2+^ were seen with 150 µM pyocyanin, but not with 300 µM 1‐hydroxphenazine. (F). ER Ca^2+^ (mean ± SEM) 8 min posttreatment in RPM2650s. Significance in bar graphs determined by one‐way ANOVA with Tukey's (B and D) or Dunnett's (*F*) posttest; *p < 0.05, **p < 0.01, ***p < 0.001.

Fitting with increases seen in Ca^2+^ signaling, only pyocyanin increased protein kinase C (PKC) activity in RPMI2650s, whereas 1‐HP did not (Figure 2A,B). PKC activation supports activation of PLC to produce diacylglycerol, which activates PKC. PLC also produces inositol trisphosphate (IP_3_) that activates the IP_3_ receptor (IP_3_R) Ca^2+^ channel in the ER. The elevation of Ca^2+^_i_ by pyocyanin in RPMI2650s was completely diminished by PLC inhibition (Figure 2C,D) and was associated with ER Ca^2+^ (Ca^2+^_ER_) release (Figure 2E,F), as measured by an ER‐targeted cameleon Ca^2+^ biosensor, D1ER. In contrast, 1‐HP increased Ca^2+^_ER_ (Figure 2E,F), though this mechanism was not further interrogated. Although our data supports possible activation of a GPCR Ca^2+^ pathway by pyocyanin, neither a Gαq inhibitor nor a T2R14 inhibitor significantly decreased Ca^2+^_i_ during pyocyanin treatment, suggesting that pyocyanin activates an unknown non‐Gαq‐coupled, non‐T2R14 receptor (Figure S1A–D). Furthermore, we saw neither downstream cAMP changes in response to pyocyanin (Figure S3G,H) nor changes in acute AMPK activity, which indicates cell stress [33] (Figure S3E,F).

Previous studies suggested that pyocyanin activates the aryl hydrocarbon receptor (AHR) that may noncanonically signal through Ca^2+^ [34, 35]. However, no Ca^2+^_i_ responses (Figure S2A,B) were seen after activation with a known AHR agonist. Similarly, the Ca^2+^_i_ was not diminished from pyocyanin activation with a known AHR inhibitor (Figure S2C,D). Nonetheless, these data together suggest possible activation of one or more receptors by pyocyanin that stimulates PLC and PKC activation and Ca^2+^_ER_ release. Thus, although the receptor driving these Ca^2+^ signals remains unknown, it could be inhibited by drugs that impact downstream PLC signaling.

Interestingly, we saw no Ca^2+^ responses with pyocyanin using protein Ca^2+^ biosensors containing a nuclear localization sequence (NLS) or nuclear export sequence (NES) to measure nuclear calcium (Ca^2+^_nuc_) or cytoplasmic calcium (Ca^2+^_cyt_), respectively (Figure S3A–D). This suggests that the Ca^2+^_i_ signals from pyocyanin using Fluo‐4 are primarily mitochondrial. Although Fluo‐4 signals typically reflect Ca^2+^_cyt_, some Fluo‐4 does load into mitochondria, and this has been used to measure Ca^2+^_mito_ in permeabilized cells, when competing signals from Ca^2+^_cyt_ are eliminated [36]. Our data suggests that pyocyanin might activate ER‐to‐mitochondrial Ca^2+^ shuttling with minimal cytoplasmic concentration increase [37], which can occur at mitochondrial‐ER contact sites, where Ca^2+^ release from the IP_3_R can transfer directly into the mitochondria [29].

### 3.2 Pyocyanin and 1‐HP Both Cause an Acute Ca^2+^‐Independent Collapse of Mitochondrial Membrane Potential and Increase in Mitochondrial Superoxide

**FIGURE 3:**
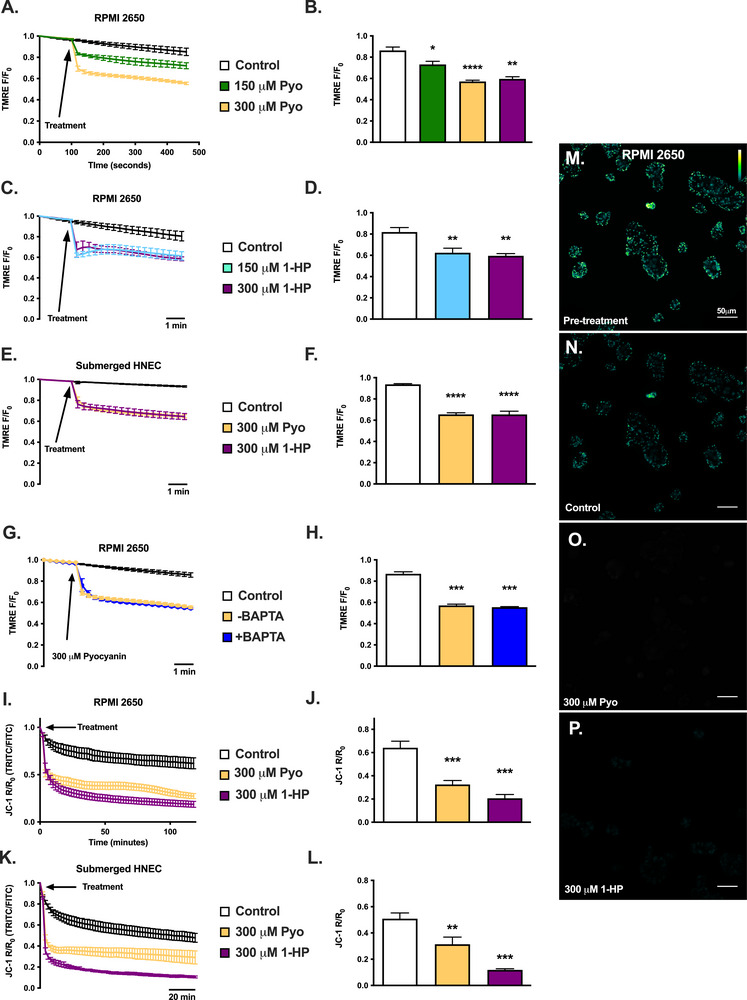
Acute mitochondrial depolarization in RPMI2650 and submerged HNEC from pyocyanin and 1‐hydroxyphenazine. (A and B) Average traces (mean ± SEM; *n* = 3 independent experiments) of tetramethylrhodamine ethyl ester (TMRE) dye fluorescence over time (A; fluorescence/fluorescence at time 0; *F/F*_0_) and mean ± SEM after 5 min (B) in RPMI2650 after treatment with HBSS (control), 150 and 300 µM pyocyanin (Pyo). A reduction in TMRE fluorescence reflects a loss of mitochondrial membrane potential. (C and D) Average traces (mean ± SEM; *n* = 4 independent experiments) of TMRE (C) and mean ± SEM after 5 min (D) in RPMI2650 after treatment with HBSS (control), 150 and 300 µM 1‐hydroxyphenazine (1‐HP). (E and F) Average traces (mean ± SEM; *n* = 3 independent experiments using cells from two patients per condition) of TMRE dye (E) and mean ± SEM after 5 min (F) in submerged primary human nasal epithelial cells (HNEC) after treatment with HBSS (control), 300 µM Pyo, and 300 µM 1‐HP. (G and H) Average traces (mean ± SEM; *n* = 4) independent experiments (G) and mean ± SEM after 5 min (H) of TMRE ± 10 µM BAPTA loading in RPMI2650 with subsequent treatment with HBSS (control) and 300 µM Pyo. (I and J) Average traces (mean ± SEM; *n* = 9 independent experiments) of JC‐1 fluorescence over time and (I) mean ± SEM after 1.5 h (J) in RPMI2650 after treatments with HBSS (control), 300 µM Pyo, and 300 µM 1‐HP. (K and L) Average traces (mean ± SEM; *n* = 3 independent experiments using cells from three patients per condition), with error bars, of JC‐1 dye fluorescence over time and (L) and mean ± SEM after 1.5 h in submerged HNEC after treatments with HBSS (control), 300 µM Pyo, and 300 µM 1‐HP. Similar to TMRE, the reduction in JC‐1 *R/R*_0_ fluorescence indicates a loss of mitochondrial membrane potential. (M–P) Representative images of JC‐1‐loaded RPMI2650s before treatment with HBSS (control, showing baseline; M) and 1.5 h post‐treatment with HBSS (N), 300 µM Pyo (O), or 300 µM 1‐HP (P). Significance in bar graphs determined by one‐way ANOVA with Dunnett's posttest: *p < 0.05, **p < 0.01, ***p < 0.001, ****p < 0.0001.

Despite the differential effects on Ca^2+^ signaling and PKC activation, mitochondrial membrane potential drastically decreased within minutes after treatment with pyocyanin or 1‐HP. We tested this using tetramethylrhodamine ethyl ester (TMRE, single wavelength) or JC‐1 (dual wavelength) mitochondrial membrane potential indicators in submerged HNECs or RPMI2650s (Figure 3). Preloading RPMI2650s with potent Ca^2+^ chelator BAPTA (1‐h incubation with 10 µM BAPTA‐AM) did not diminish mitochondrial membrane depolarization (Figure 3G,H), indicating that this depolarization is Ca^2+^‐independent. Pyocyanin that was extracted from planktonic *P. aeruginosa* cultures from lab and clinical strains also activated robust mitochondrial membrane depolarizations (Figure S4A–D) and superoxide production in RPMI2650 cells and primary HNECs (Figure S4E–I). Conditioned media also increased superoxide production (Figure S4G). Further supporting a lack of involvement of the AHR in the pyocyanin responses observed here, an AHR agonist had no effect on mitochondrial membrane potential (Figure S5A,B), suggesting AHR activation does not mimic the effects of pyocyanin or 1‐HP.

**FIGURE 4:**
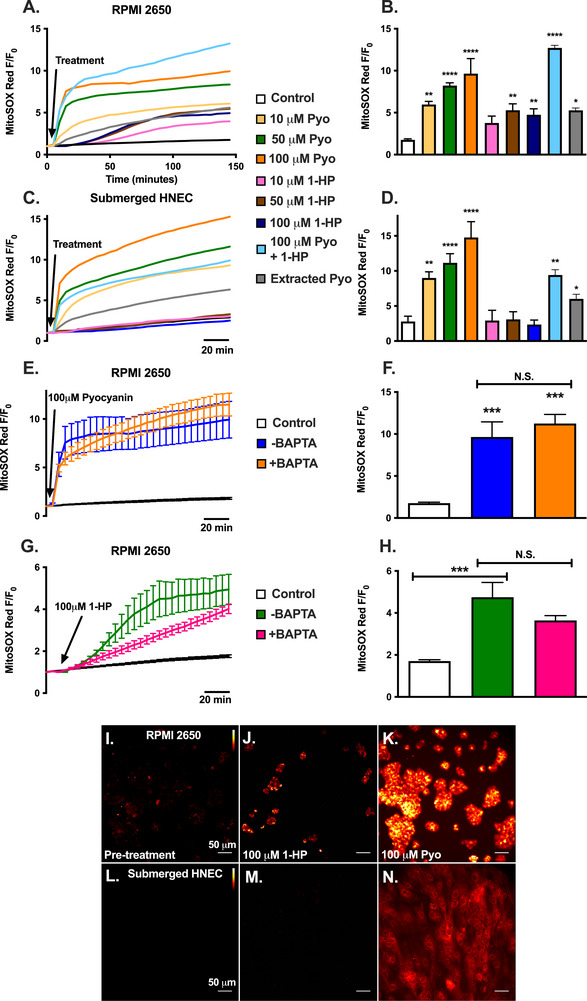
Pyocyanin and 1‐hydroxyphenazine induce superoxide production in RPMI2650, but only pyocyanin induces superoxide production in submerged HNEC. (A and B) Average traces (mean; *n* = 9 independent experiments) of MitoSOX Red fluorescence over time (A; fluorescence/fluorescence at time 0; *F/F*_0_) and mean ± SEM after 2 h (B) in RPMI2650 after treatment with HBSS (control), 10 µM pyocyanin (Pyo), 50 µM Pyo, 100 µM Pyo, 10 µM 1‐hydroxyphenazine (1‐HP), 50 µM 1‐HP or 100 µM 1‐HP, 100 µM Pyo + 1‐HP, or extracted pyocyanin from ATCC 27853 (18.2 µM). An increase in MitoSOX Red fluorescence indicates an increase in superoxide production. (C and D) Average traces (mean; *n* = 3 independent experiments using cells from ≥3 patients per condition) of MitoSOX Red fluorescence over time (*C*) and mean ± SEM after 2 h (D) in submerged primary human nasal epithelial cells (HNEC) after treatment with HBSS (control), 10 µM Pyo, 50 µM Pyo, 100 µM Pyo, 10 µM 1‐HP, 50 µM 1‐HP, or 100 µM 1‐HP, or extracted pyocyanin from ATCC 27853 (33.9 µM). (E–H). Average traces (mean ± SEM; *n* = 3 independent experiments) (E and G) and mean ± SEM after 2 h (F and H) of TMRE ± pre‐loading with 10 µM BAPTA‐AM in RPMI2650s after treatment with HBSS (control), pyocyanin, or 1‐HP as indicated. (I–N) Representative images of MitoSOX Red loaded RPMI2650 (I–K) and submerged HNEC (L–N) at baseline or 2 h posttreatment with 100 µM 1‐HP, or 100 µM Pyo, as indicated. Significance in bar graphs determined by one‐way ANOVA with Dunnett's or Bonferroni posttest; *p < 0.05, **p < 0.01, ***p < 0.001, ****p < 0.0001; N.S., not significantly different.

Notably, pyocyanin (10 µM) activated stronger increases in mitochondrial superoxide production compared with 1‐HP in RPMI2650s and submerged HNECs (Figure 4A–D). Extracted pyocyanin from ATCC27853 also enhanced superoxide production. Unlike the Ca^2+^ response described in Figure 1, the combination of pyocyanin and 1‐HP did not negate the superoxide production. However, the combination of pyocyanin and 1‐HP also did not enhance superoxide production (Figure 4A–D), suggesting they activate the same pathway rather than complementary pathways. Pre‐treatment with 10 µM BAPTA (as in Figure 3) showed no significant decrease in pyocyanin‐stimulated superoxide production in RPMI2650 and only slightly hindered 1‐HP‐stimulated superoxide production after 2 h in RPMI2650s (Figure 4E–H), suggesting that the effects of phenazines on mitochondrial membrane potential are largely Ca^2+^‐independent. Representative images of MitoSOX fluorescence increases in RPMI 2650s and primary HNECs are shown in Figure 4I–N.

### 3.3 Enhanced Susceptibility to Phenazine‐Induced Cytotoxicity in Cancer Cell Lines May Involve Altered Modulation of ATF6 and Other Genes Involved in ER Stress and Protein Homeostasis

**FIGURE 5:**
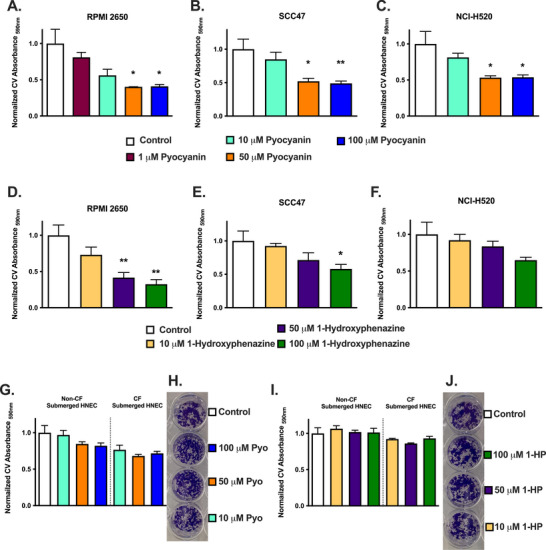
Pyocyanin and 1‐hydroxyphenazine reduce viability of RPMI2650, SCC‐47, and NCI‐H520 carcinoma cells but not submerged HNEC. (A–F) Crystal Violet (CV) absorbance in RPMI2650s (A and D), SCC‐47 (B and E), and NCI‐H520 (C and F) after 24‐h treatment with media, pyocyanin (A–C), or 1‐hydroxyphenazine (D–F) as indicated. A decrease in CV absorbance indicates a reduction in the number of cells and thus reflects cell viability. *n* ≥ 3 independent experiments per conditions/cell type. (G–J) CV absorbance (G and I) and representative images (H and J) from submerged non‐CF and CF HNECs after 24‐h treatment with media, pyocyanin, or 1‐hydroxyphenazine, as indicated. *n* = 3 independent experiments using cells from three patients per condition. Significance in bar graphs determined by one‐way ANOVA with Dunnett's posttest; *p < 0.05, **p < 0.01, ***p < 0.001.

Both pyocyanin and 1‐HP decreased viability in squamous carcinoma cell lines RPMI2650, SCC‐47, and NCI‐H520, but not in submerged HNECs from either CF or non‐CF individuals, at 24 h, shown by crystal violet staining (Figure 5). HNECs appeared to be resistant to the cytotoxic effects of pyocyanin and 1‐HP. Cytotoxic effects in RPMI2650 were independent of activation of apoptotic executioner caspases‐3 and ‐7 (Figure S6A–E), which also did not exhibit increased expression with pyocyanin or 1‐HP treatment (Figure S7B,C). We began to look for other gene expression changes that could explain the discrepancy between cancer and primary cell toxicity. Upregulation of antioxidant gene *HMOX‐1* was observed in RPMI2650s (Figure S7) and possibly HNECs (Figure S8). *IL1B* was downregulated in pyocyanin‐treated RPMI2650s (Figure S9). Although various cytokines, including *IL6, IL1B*, and *TNF*, showed a trend toward upregulation in submerged HNEC, none of these changes were significant (Figure S10). Overall, we did not see major changes in cytokine or antioxidant signaling genes between HNECs and RPMI2650s that would likely explain the differential cytotoxicity.

**FIGURE 6:**
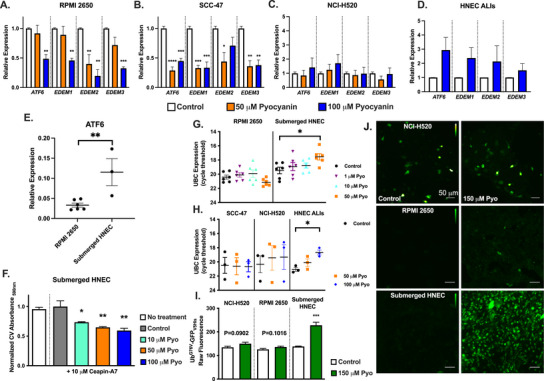
Pyocyanin inhibits ER stress response in some squamous cell carcinoma lines but induces a response in primary HNECs. (A–D) *ATF6, EDEM1, EDEM2*, and *EDEM3* expression in RPMI2650 (A), SCC‐47 (B), NCI‐H520 (C), or HNEC ALIs (D). Statistically significant decreases in all genes shown were seen in RPMI2650 and SCC‐47 by one‐way ANOVA with Dunnett's posttest (*n* ≥ 3 independent experiments, **p < 0.01, ***p <0.001, ****p<0.0001). In either NCI‐H520, expression was unchanged, whereas HNEC ALIs showed a trend toward increased gene expression, though not statistically significant. (E) Baseline *ATF6* expression (mean ± SEM; *n* ≥ 3 independent experiments) in RPMI2650 versus submerged HNEC. Submerged HNEC was statistically significant compared to RPMI2650 (unpaired *t*‐test, **p < 0.01). (F) Crystal Violet (CV) absorbance in submerged HNEC (mean ± SEM; *n* ≥ 3 independent experiments) after 24‐h treatment with ceapin‐A7 and pyocyanin as indicated. All concentrations of pyocyanin treated with ceapin‐A7 were statistically significant compared to control (one‐way ANOVA with Bonferroni's posttest). (G and H) Raw cycle threshold (Ct) values for Ubiquitin C (*UBC*) in RPMI2650 or submerged HNEC (*n* = 6) after 24‐h treatment with media (control), 1, 10, or 50 µM pyocyanin (Pyo) (G) or in SCC‐47, NCI‐H520, or HNEC ALI (*n* = 3) after 24‐h treatment with media (control), 50 µM, or 100 µM Pyo (H). Significance in G and H by one‐way ANOVA with Dunnett's posttest (n ≥ 3 independent experiments, *p < 0.05). (I) Raw fluorescence values (mean ± SEM; *n* = 4 independent experiments using cells from three patients per condition in primary HNEC) of NCI‐H520, RPMI2650, or submerged HNECs transfected with Ub^G76V^‐GFP_V5His_ (a GFP‐tagged mutated ubiquitin proteasome substrate) after 4‐h treatment with media (control) or 150 µM Pyo. Only submerged HNEC had statistically significant increases of ubiquitinated Ub^G76V^‐GFP_V5His_, whereas neither cancer cell line (NCI‐H520 or RPMI2650) showed increases (unpaired *t*‐test; ***p < 0.001). (J) Representative images of NCI‐H520s, RPMI2650s, and submerged HNECs transfected with Ub^G76V^‐GFP_V5His_ after 4 h treatments with media only (vehicle control) or 150 µM Pyo. HNEC, human nasal epithelial cells.

We next hypothesized that pyocyanin depletion of Ca^2+^_ER_ and/or reactive oxygen species (ROS) production might differentially alter expression of genes involved in the ER unfolded protein response (UPR^ER^) and ER‐associated degradation (ERAD). Ca^2+^_ER_ is required for proper folding of proteins that transit the secretory pathway through the ER [38], and oxidative stress can also damage ER proteins and cause ER stress [39]. A previous study in renal tubular cells showed that pyocyanin induces *ATF6* transcriptional activity in NRK‐52E cells [16]. *ATF6* is an important transducer of UPR^ER^, and we hypothesized that this may be important to cell survival during pyocyanin treatment. *ATF6* promotes survival of buccal squamous carcinoma [40] and other cancers [41], whereas *ATF6* knockdown reduces oral squamous carcinoma cell proliferation by inhibiting autophagy [42]. Activation of autophagy in host cells in response to pyocyanin was suggested to reduce bacterial burden and infection mortality in mice [43]. We noted that *ATF6* expression was higher at baseline in submerged HNECs compared to RPMI2650s (Figure 6E), suggesting differential potential to respond to ER stress. Surprisingly, we found that pyocyanin induces significant downregulation of *ATF6*, *EDEM1* (a known transcriptional target of *ATF6*), *EDEM2*, and *EDEM3* in RPMI2650s and SCC‐47s but not in NCI‐H520s and HNEC air–liquid interface cultures (ALIs) (Figure 6A–D). A preliminary analysis suggested potentially longer survival in patients with low tumor *ATF6* expression in head and neck (HNSCC) and lung squamous cell carcinomas (LSCC) cells (Figure S11A), whereas other UPR^ER^ and ERAD markers showed no significant differences (Figure S11B–D). Additionally, preliminary stage plot analysis of HNSCC and LSSC showed upregulation of ATF6 expression in later‐stage cancers (Figure S11E,F). Although further analyses need to be done to understand the differential toxicity between cancer and primary cells, the downregulation of *ATF6* by phenazines in cancer cells may enhance pyocyanin cytotoxicity by reducing the cancer cells ability to adapt to ER stress. Thus, from a host–pathogen interactions standpoint in the context of CF‐CRS, our data suggest that phenazine responses in primary cells may markedly differ from cell lines derived from tumors.

Further supporting an ability of primary HNECs to better adapt to pyocyanin‐induced protein homeostasis challenges, we noted that ubiquitin C (*UBC*) expression was upregulated in submerged HNECs and ALIs in response to pyocyanin (Figure 6G). *UBC* expression is increased during ER stress to provide increased ubiquitin capacity to handle damaged and/or unfolded proteins [44, 45]. In contrast, *UBC* expression was largely unchanged in the cancer cell lines (RPMI2650, SCC‐47, or NCI‐H520) (Figure 6H). We saw alterations in the levels of a ubiquitin‐substrate biosensor protein in HNECs after treatment with pyocyanin, whereas cancer cells (RPMI2650 and NCI‐H520) showed no changes (Figure 6I,J). Together, our data suggest that reduced expression of genes involved in protein homeostasis might explain the cytotoxic effects seen in cancer cell lines, whereas upregulation or baseline maintenance of these same genes in HNECs may allow adaptation to reduce cytotoxicity.

### 3.4 Effects of Pyocyanin on Mitochondrial Ca^2+^, Membrane Potential, ROS, and Cilia Function in HNEC ALIs Show Enhanced ROS Production and Ciliotoxicity in CF Cells

**FIGURE 7:**
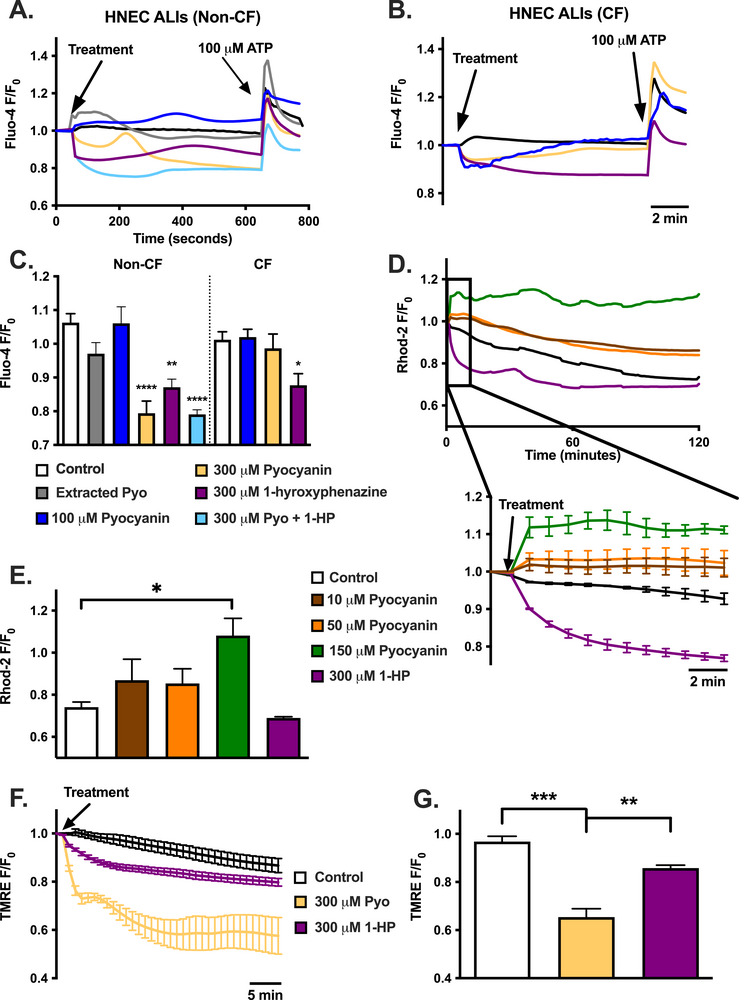
Pyocyanin and 1‐hydroxyphenazine do not increase cytosolic Ca^2+^ in primary HNEC ALIs, but pyocyanin increases mitochondrial calcium, and both phenazines decrease mitochondrial membrane potential. (A and B) Average traces (mean ± SEM) of Fluo‐4 Ca^2+^_i_ (fluorescence/fluorescence at time 0; *F/F*_0_) in HNEC ALIs from non‐CF (A) and CF (B) individuals. (C) Ca^2+^_i_ (Fluo‐4 *F/F*_0_; mean ± SEM) 10 min posttreatment in non‐CF or CF HNEC ALIs with 0 µM (HBSS vehicle control), 100 µM, or 300 µM pyocyanin, or 300 µM 1‐hydroxyphenazine, or extracted pyocyanin from ATCC 27853 (18.2 µM) (non‐CF only), or 300 µM pyocyanin + 1‐hydroxyphenazine (non‐CF only). No treatment was statistically different from control; *n* ≥ 3 independent experiments. (D) Average traces (mean ± SEM) of Rhod‐2 Ca^2+^_mito_ in non‐CF HNEC ALIs, and (E). Ca^2+^_mito_ (mean ± SEM) 90 min posttreatment in non‐CF HNEC ALIs with 0 µM (HBSS vehicle control), 10 µM, 50 µM, or 100 µM pyocyanin, or 300 µM 1‐hydroxyphenazine; *n* ≥ 3 independent experiments. 150 µM pyocyanin (***) was significant, compared to control, at 1‐min posttreatment. (F) Average traces (mean ± SEM) of TMRE and (G). Mean ± SEM after 10 min in non‐CF HNEC ALIs after treatment with 0 µM (HBSS vehicle control), 300 µM pyocyanin, or 300 µM 1‐hydroxyphenazine; *n* ≥ 3 independent experiments. Significance in bar graphs determined by one‐way ANOVA with Dunnett's (C and E) or Tukey's (G) posttest; *p < 0.05, **p < 0.01, ***p < 0.001, ****p < 0.0001.

**FIGURE 8:**
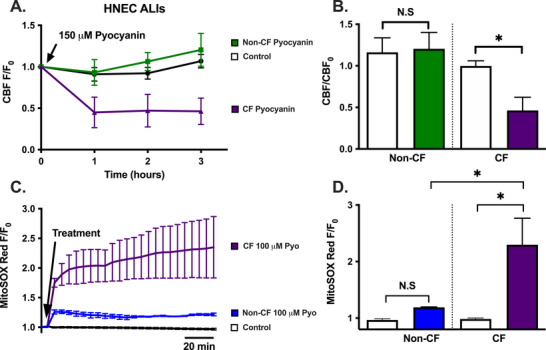
Pyocyanin decreases ciliary beat frequency (CBF) and induces superoxide production in HNEC ALIs. (A and B) Average traces (mean ± SEM; A) of CBF (frequency/frequency at time 0; *F/F*_0_) in non‐CF versus CF HNEC ALI and bar graph (B) of CBF (mean ± SEM) 4 h after treatment with HBSS (control) or 150 µM pyocyanin (*n* ≥ 3 independent experiments). Only 150 µM pyocyanin was statistically significant at 4 h posttreatment in CF cells when compared to control by one‐way ANOVA. (C and D) Average traces (mean ± SEM; C) of MitoSOX Red fluorescence over time (fluorescence/fluorescence at time 0; *F/F*_0_) and bar graph (mean ± SEM; D) of fluorescence after 90 min in HNEC ALI (non‐CF versus CF) after treatment with HBSS (control) or 100 µM pyocyanin (*n* ≥ 3 independent experiments). Significance in (B) and (D) by one‐way ANOVA with Bonferroni's posttest with paired comparisons (non‐CF control versus non‐CF pyocyanin and non‐CF pyocyanin vs. CF pyocyanin); *p < 0.05; N.S., not significantly different. ALI, air–liquid interface; CBF, ciliary beat frequency; HNEC, human nasal epithelial cells.

Interestingly, pyocyanin did not induce robust Ca^2+^_i_ increases observed with Fluo‐4 in either non‐CF or CF HNECs ALIs, differing from submerged HNECs and RPMI2650s (Figure 7A–C). We found that pyocyanin (at 300 µM but not 100 µM), 1‐HP (at 300 µM), or a combination of pyocyanin plus 1‐HP appeared to lower Ca^2+^_i_ (Figure 7A–C). Extracted pyocyanin did not lower Ca^2+^, but the concentration of the extracted pyocyanin was an order of magnitude below 300 µM. However, Ca^2+^_mito_ did increase in ALIs with pyocyanin, as measured via Rhod‐2. Ca^2+^_mito_ decreased with 1‐HP (Figure 7D,E). It may be that the organization of ER/mitochondrial contact points in polarized ALIs allows more direct ER‐to‐mitochondrial Ca^2+^ transfer, resulting in reduced Ca^2+^_cyt_ responses but intact Ca^2+^_mito_ responses. Nonetheless, pyocyanin and 1‐HP both induced mitochondrial membrane depolarization in ALIs, although the depolarization with pyocyanin was much greater (Figure 7F,G). We noted differences between CF and non‐CF HNEC ALI responses with ciliary beat frequency (CBF), which decreased after 4 h of exposure to 150 µM pyocyanin in CF cells but not non‐CF cells (Figure 8A,B). Notably, pyocyanin induced much higher superoxide production in CF HNEC ALIs compared with non‐CF ALIs (Figure 8C,D). Excess ROS production is well documented to impair cilia function [46, 47, 48, 49] and thus we hypothesize that the excess ROS in CF cells leads to enhanced ciliotoxicity.

## 4 Discussion

We initially investigated Ca^2+^ signaling, which was previously linked to pyocyanin [13, 19]. Although Ca^2+^ is essential for oxidative phosphorylation and mitochondrial metabolism, excessive Ca^2+^ influx into mitochondria can cause Ca^2+^ overload and excessive ROS production [7, 25], leading to mitochondrial dysfunction and, frequently, activation of apoptosis [17]. We hypothesized that Ca^2+^ would be involved in mitochondrial toxicity with both pyocyanin and 1‐HP. However, we found that only pyocyanin induces primarily Ca^2+^_mito_ responses, whereas 1‐HP had no detectable effects on either cytoplasmic or mitochondrial Ca^2+^. The lack of cytoplasmic/global Ca^2+^ responses observed with Fluo‐4 but robust Ca^2+^_mito_ responses suggests that pyocyanin activates ER Ca^2+^ release in close proximity to ER‐mitochondria contact points [18], where direct Ca^2+^ transfer between the two organelles can sometimes occur without robust cytoplasmic Ca^2+^ increases [29, 50], which has been termed ER‐mitochondrial Ca^2+^ shuttling. This has been reported in neurons and other cell types with GPCR activation [37]. However, it is a relatively unexplored phenomenon in the context of the airway epithelium. Previous studies that have reported cytoplasmic Ca^2+^ increases with pyocyanin [13, 19] may have used indicator dye loading conditions or cell types that promoted significant accumulation of dyes into the mitochondria, which can sometimes occur. This has even been exploited experimentally to measure Ca^2+^_mito_ [36]. We believe that our more rigorous, targeted Ca^2+^ measurements here support a minimal cytoplasmic but robust mitochondrial elevation of Ca^2+^ with pyocyanin.

Because (1) pyocyanin also increased PKC activity, (2) the Ca^2+^ response was diminished with a PLC inhibitor, and (3) ER Ca^2+^ stores were decreased, we hypothesize that pyocyanin activates a GPCR. However, the responses did not appear to be dependent on T2Rs, and future work is needed to identify the host pyocyanin receptor. Although rare, direct small molecule activation of PLC has also been reported [51, 52]. It also remains possible that pyocyanin might directly activate PLC, bypassing a GPCR to stimulate both IP_3_ production to cause Ca^2+^ release and diacylglycerol production to activate PKC. Because of the localized nature of the Ca^2+^ response, which appears to be restricted to ER/mitochondrial contact points, we speculate that a localized GPCR is the more likely target. However, pyocyanin activation of a specific PLC isoform that is likewise spatially restricted could also explain these results. It is possible that more than one unknown target of pyocyanin, and likely 1‐HP, exists in airway epithelial cells, possibly including TLRs and/or the aryl hydrocarbon receptor (AHR) [53], though the AHR was not activated in RPMI2650s and did not appear to be responsible for any effects observed here. Although we were not successful in identifying the receptor in this study, the information presented here regarding the signal pathways activated and their localization may be useful for future efforts to identify the pyocyanin receptor.

Despite the differential Ca^2+^ effects of pyocyanin and 1‐HP, mitochondrial membrane potential decreased similarly with either phenazine. Furthermore, pyocyanin greatly increased superoxide production in both RPMI2650s and submerged HNECs, whereas 1‐HP induced smaller increases (RPMI2650) or had no effect (submerged HNEC). Although we expected this to be related to the differential Ca^2+^_mito_ responses, the mitochondrial ROS production was surprisingly Ca^2+^ independent. Pyocyanin may also activate EGFR‐PI3K‐AKT/MEK1/2‐ERK1/2 MAPK pathways in A549 cells to increase nuclear levels of the antioxidant transcription factor Nrf‐2 [54, 55]. However, EGFR and Akt pathways are typically pro‐survival rather than pro‐death pathways [56]. Notably, an early study reported that, although both 1‐HP and pyocyanin inhibited mitochondrial respiration in intact cells, only 1‐hydroxyphenzine inhibited respiration of isolated liver mitochondria [57]. These authors hypothesized that inhibition of mitochondrial function by pyocyanin requires intracellular demethylase conversion of pyocyanin to 1‐HP. Early studies also reported that 1‐HP interacts with ubiquinone‐cytochrome b within the mitochondrial electron transport chain [58, 59]. We speculate that the Ca^2+^ responses observed here are due to pyocyanin activation of an unknown GPCR, whereas the mitochondrial depolarization and reduced viability of cell lines we observe are due to intracellular conversion of pyocyanin to 1‐HP, explaining the similar responses observed for membrane potential and cell proliferation. However, this is speculation, and the greater levels of mitochondrial superoxide observed with pyocyanin compared with 1‐HP suggest not all of the mitochondrial dysfunction can be due to pyocyanin conversion to 1‐HP as they are not equivalent.

Both pyocyanin and 1‐HP displayed similar decreases in cell viability in RPMI2650s and other cancer cells. However, HNECs cultured identically (submerged conditions) did not die after 24 h. This may be due to differences seen here in UPR^ER^ and ERAD markers in cells derived from cancer versus normal tissue. Pyocyanin seemed to reduce UPR^ER^ and ERAD markers in cancer cells but not in non‐cancer primary HNECs. This may indicate an alteration of protein homeostasis in some cancer cells. Alternatively, a recent study showed that primary airway epithelial cells may have altered electron transport due to elevated mitochondrial uncoupling proteins [60]. This likely protects against oxidative stress but also decreases the percentage of mitochondrial ATP generation [60]. If primary HNECs are indeed less reliant on mitochondrial respiration for ATP, it might also protect them against the impairment of mitochondrial electron transport by phenazines. Nonetheless, the greater sensitivity of RPMI2650s and other cancer cells to phenazine‐induced cytotoxicity underscores the necessity to study the effects of bacterial phenazines in primary non‐cancerous cells to better understand their effects in an immune context. Studying host responses to phenazines in cancer cell lines may not reflect the responses that occur in primary differentiated cells in vivo.

Finally, our data reveal a potentially important insight into CF and CF‐CRS pathophysiology. Pyocyanin enhanced mitochondrial ROS production in HNEC ALIs to a much greater degree than 1‐HP in HNECs, suggesting that pyocyanin is the more damaging virulence factor and that somehow altering *P. aeruginosa* to produce more 1‐HP and less pyocyanin may reduce ROS production. Moreover, HNEC ALIs from CF‐CRS tissue showed enhanced sensitivity to pyocyanin for ROS production compared with non‐CF ALIs. We hypothesize this may be due to previously reported downregulation of antioxidant transcription factor Nrf‐2 in CF [61, 62, 63], which may decrease cellular responses to oxidative stressors [64]. Enhanced ROS production likely causes the reduction in CBF observed only in CF‐CRS cells.

A limitation of our study is that we are using purified pyocyanin as well as extracted pyocyanin from *P. aeruginosa*. A better understanding of the role of pyocyanin in infection likely requires studies of infection models using primary nasal ALIs or animals. Live or heat‐killed *P. aeruginosa* were not used here, as gram‐negative bacteria contain other factors that can influence Ca^2+^ signaling, including LPS [65] and flagellin [66]. Infection models using pyocyanin‐deficient *P. aeruginosa* may be challenging because pyocyanin is regulated by the LasR/RhlR quorum‐sensing pathways at multiple steps in their biosynthesis [67, 68]. These quorum‐sensing pathways also regulate other virulence factors like elastase that are important for infection [69], and thus specific genetic targeting of pyocyanin is needed to appreciate the specific contribution of pyocyanin among the milieu of *P. aeruginosa* virulence factors produced during infection. Likewise, work is still needed to understand how Ca^2+^ signaling fits into *P. aeruginosa* infection. We recently found that *P. aeruginosa* 3‐oxo‐C12HSL‐induced is largely Ca^2+^ dependent, but a clearer picture of how host cell Ca^2+^ responses contribute to *P. aeruginosa* infection is needed. This is complicated by the many pathways by which *P. aeruginosa* can activate Ca^2+^ signals or even inhibit host receptors like PAR‐2 that signal through Ca^2+^ [70]. Nonetheless, our data here reveal several molecular details of pyocyanin's effects on nasal epithelial cells. We show potential differences that may exist in CF cells that may leave them more vulnerable to *P. aeruginosa*. We also demonstrate molecular mechanisms altered in cancer‐derived cell lines that make them insufficient for studying the effects of phenazines on host cells.

## 5 Conclusion

Together, these data suggest that acute Ca^2+^ signaling is not required for reduction in mitochondrial function with *P. aeruginosa* phenazines. Likewise, the acute reductions in mitochondrial function coupled with superoxide production are not likely the only mechanisms for the decreased cell viability in cells exposed to phenazines. Similar mitochondrial depolarization and ROS production were observed in both HNECs and squamous cell carcinoma cell lines, but only the cancer‐derived cells died, possibly due to downregulation in UPR^ER^ and ERAD markers in cancer cells. This supports a need for using primary cells to understand host–pathogen interactions mediated by pyocyanin. Although the exact mechanism(s) of cell death in the cell lines with pyocyanin and 1‐HP remain to be determined, the survival of primary cells and altered expression of stress genes indicate that ER stress and protein homeostasis may play a crucial role in host cell defense against pyocyanin toxicity. Nonetheless, the decrease in mitochondrial function and cell viability induced by both pyocyanin and 1‐HP is not likely through a T2R/Ca^2+^‐dependent pathway. Furthermore, CF cells may have a higher sensitivity towards pyocyanin, with CF ALIs exhibiting increased ROS and higher ciliotoxicity compared with non‐CF cells.

## Funding

This study was supported by National Institutes of Health Grants AI167971 and HL181155 to R.J.L., N.D.A., and J.N.P. and HL168060 to R.J.L., as well as Cystic Fibrosis Foundation Grant LEE25G0 to R.J.L. and an American Academy of Otolaryngology AAO‐HNSF Resident Research Award to N.F.D.

## Conflicts of Interest

The authors declare no conflicts of interest.

## Supporting information




**Supporting File 1**: alr70084‐sup‐0001‐SuppMat.pdf.

**Supporting File 2**: alr70084‐sup‐0002‐SuppMat.xlsx.
