# Supplementary material for: Mechanisms of Mitochondrial Toxicity and Cytotoxicity Caused by Pseudomonas aeruginosa Pyocyanin in Human Nasal Epithelial Cells
Source: Int Forum Allergy Rhinol. 2025 Dec 19;16(5):451–66. doi: 10.1002/alr.70084 (PMC13147224; doi:10.1002/alr.70084)
Supplement: Supplementary file 1 — Supporting File 1: alr70084‐sup‐0001‐SuppMat.pdf. [file ALR-16-451-s001.pdf]

Supplemental Materials and Methods

Reagents and Experimental Methods

Hanks' balanced salt solution (HBSS) was used in cell imaging experiments, containing 137 mM NaCl, 5 mM KCl, 400 µM KH_2PO_4, 300 µM Na_2HPO_4, with or without 5.6 mM glucose, 1.8 mM $CaCl_2$, 1.5 mM $MgCl_2$, 20 mM HEPES, pH 7.4. HBSS containing 5.6 mM glucose is hereafter referred to as HBSS with glucose, and HBSS without 5.6 mM glucose referred to as HBSS without glucose. Crystal Violet (CV) solution was used in cell viability assays which contained 0.1% CV in DI water with 10% acetic acid. BAPTA, CH 223191, ionomycin, lupulone, pyrene, pyocyanin, thapsigargin, U73122, U73343, VBIT-12, YM-254890, and 1-hydroxyphenazine were from Cayman Chemical. ATP, Benzethonium Chloride, and Isoproterenol were from Sigma-Aldrich. 6-methoxyflavonone (6-MF) was from Vitas-M Laboratory. For experiments with cell lines, each replicate was an independent experiment using a separate cell culture. For primary HNECs, independent replicates used cells from different individual patients.

Live cell imaging of cytosolic Ca^{2+} (Ca^{2+}_{cyt}), mitochondrial Ca^{2+} (Ca^{2+}_{mito}), ER Ca^{2+} (Ca^{2+}_{ER}), and PKC activity

Submerged HNEC or RPMI2650 cells were loaded with 5 µM Fluo-4 AM (AAT-bioquest; Pleasanton, California, USA) in HBSS with glucose for 1 hour in the dark at room temperature (RT). HNEC ALIs were loaded apically with 10 µM Fluo-4 AM in HBSS without glucose, containing 295 µg/mL Probenecid and 394 µg/mL Pluronic® F127 for 2 hours at RT in the dark. For submerged cells (RPMI2650 or submerged HNEC), Ca^{2+}_{cyt} was imaged using a Nikon Eclipse TS100 microscope (20x 0.75 NA objective), FITC filters (Chroma Technologies), QImaging Retiga R1 camera (Teledyne; Tucson, Arizona, USA), MicroManager software, and XCite 120 LED Boost (Excelitas Technologies; Mississauga, Canada). For HNEC ALIs, Ca^{2+}_{cyt} was imaged using an Olympus IX-83 microscope (10x 0.40 NA objective), FITC filters (Chroma Technologies), Orca Flash 4.0 sCMOS camera (Hamamatsu, Tokyo, Japan) and MetaFluor (Molecular Devices; Sunnyvale, CA USA)

For Ca^{2+}_{mito}, Ca^{2+}_{ER}, or PKC activity, submerged HNECs or RPMI2650s were transfected with pCMV-Mito-LAR-Geco1.2 (Addgene Plasmid #61245;[1]), pcDNA-D1ER (Addgene Plasmid #36325;[2]), or CMV-ExRaiCKAR (Addgene Plasmid #118409;[3]) respectively, using Lipofectamine 3000 reagents (ThermoFisher Scientific) per the manufacturer's instructions 48 hours prior to imaging. Ca^{2+}_{mito} was imaged with an Olympus IX-83 microscope (20x 0.75 NA objective), TRITC filters (Chroma Technologies), Orca Flash 4.0 sCMOS camera (Hamamatsu, Tokyo, Japan) and MetaFluor (Molecular Devices; Sunnyvale, CA USA). Ca^{2+}_{ER} or PKC activity was imaged as described above with the Olympus IX-83 microscope system, but with CFP and YFP, or TRITC and FITC filters (Chroma Technologies) respectively. For Ca^{2+}_{mito} in HNEC ALI, cells were loaded with 5 µM Rhod-2 AM (AAT-bioquest;[4, 5]) for 18 hours in the dark at 4°C. After loading, ALIs were incubated on the basolateral side only in F-12K Media (Gibco) at 37°C for 1 hour. Cells were imaged in HBSS without glucose on the apical side and HBSS with glucose on the basolateral side. Imaging occurred on the same system as HNEC ALI Ca^{2+}_{cyt} but with a standard TRITC filter set and MetaMorph imaging software (Molecular Devices).

Live cell imaging of Ca^{2+}_{cyt} with inhibitors, nuclear Ca^{2+} (Ca^{2+}_{nuc}), AMPK activity, and cAMP activity

RPMI2650s were loaded with Fluo-4 AM as described above and co-loaded with

either 10 μM U73343 (inactive PLC inhibitor) or U73122 (active PLC inhibitor), 10 μM YM-254890 (Gαq inhibitor), 100 μM 6-methoxyflavonone (6-MF), 500 μM LF1 (both T2R14 inhibitors), or 100 μM CH 223191 (AHR inhibitor). Imaging occurred with the same imaging system as from Ca^{2+}_{cyt}.

For Ca^{2+}_{nuc} (localized or export), AMPK-activated protein kinase A reporter (AMPKAR) activity, or cyclic AMP (cAMP) activity, RPMI2650 were transfected with CMV-NLS-R-Geco (Addgene Plasmid #32462;[6]), CMV-NES-R-Geco1 (created in-house from Addgene Plasmid #32444), AMPKAR (Addgene Plasmid #35097;[7]), or Flamindo2 (Addgene Plasmid #73938;[8]) respectively, using Lipofectamine 3000 reagents (ThermoFisher Scientific) 48 hours prior to imaging. CMV-NES-R-Geco1 was created by inserting NES into CMV-R-Geco1 (Addgene Plasmid #32444;[6]). Ca^{2+}_{nuc} or AMPK activity was imaged with the same imaging system as described above with Ca^{2+}_{mito} or Ca^{2+}_{ER}, while cAMP activity was imaged as described above but with FITC filters (Chroma Technologies).

Mitochondrial membrane potential, superoxide production, cell viability, and apoptosis measurement

For acute single-wavelength mitochondrial membrane potential measurement, HNEC (submerged or ALI) and RPMI2650 cells were loaded for 15 minutes at RT in the dark with tetramethylrhodamine ethyl ester (TMRE) dye and Hoechst dye prepared as described in the Multi-Parameter Apoptosis Assay Kit (Cayman Chemical; Ann Arbor, MI, USA). Cells were imaged with TRITC filters (Chroma Technologies) as described above with the Olympus IX-83 microscope system and 20x lens (for submerged HNEC and RPMI2650) or 10x lens (for ALI). For longer-term mitochondrial membrane potential measurement, submerged HNEC and RPMI2650 cells were loaded for 15 minutes at 37°C and 5% CO_2 with 1.5 μM JC-1 dye (Cayman Chemical), which allowed ratiometric green-red imaging. Imaging occurred as described above with the Olympus IX-83 microscope system, but with TRITC and FITC filters (Chroma Technologies) and MetaMorph (Molecular Devices).

For monitoring mitochondrial superoxide production, HNEC (submerged or ALI) and RPMI2650 cells were loaded for 30 minutes at 37°C and 5% CO_2 with 0.5 μM MitoSOX™ Red dye (Life Technologies; Carlsbad, CA, USA). Imaging occurred as described above with JC-1 imaging, but with TRITC filters (Chroma Technologies).

For crystal violet cell viability assays, submerged HNEC and RPMI2650 were treated, in their respective culture medias, for 24-hours. Cells remaining on plates were stained with the CV solution described above, then washed with DI water, and allowed to dry for 6-24 hours at RT. Stains were dissolved in 30% acetic acid in DI water and absorbance was measured in a Spark 10M Multimode Microplate Reader (Tecan; Männedorf, Switzerland) at 590 nm.

For apoptosis measurement, RPMI2650 were loaded with CellEvent caspase 3/7 dye (ThermoFisher Scientific) in media with control (just media), 100 μM 1-hydroxyphenazine, 100 μM pyocyanin, or 240 μM lupulone for 21 hours. Imaging occurred as described above with JC-1 imaging, but with FITC filters (Chroma Technologies). Images were taken from ≥3 random locations per treatment group.

Ciliary Beat Frequency (CBF)

To measure ciliary beat frequency, HNEC ALIs were equilibrated on the temperature control plate set at 37°C for at least 15 minutes. Equilibration occurred in HBSS without glucose on the apical side, and HBSS with glucose in the basolateral side. CBF was measured using Sisson-Ammons Video Analysis (SAVA) software,[9] a Nikon

Diaphot microscope (Modulation Optics Inc. HMC 20x LWD 0.4NA 160/0-2 objective), and Basler acA1300-200um Camera (Ahrensburg, Germany) with a Nikon 0.7x DXM Lens (Tokyo, Japan), and Heating Insert M24 2000 EC (Pecon; Erbach, Germany). ALI inserts were treated apically, and CBF was recorded for 2.5 seconds each in 5-10 random locations per insert (averaged) every hour.

Reverse transcription quantitative Polymerase Chain Reaction (RT-qPCR) expression of endoplasmic reticulum (ER) genes, cytokines, mitochondrial stress, and pro-apoptosis markers

RPMI2650 and HNEC (submerged or ALI) were treated, for either 16 or 24 hours, with various concentrations of pyocyanin or 1-hydroxyphenazine (50 μM, 10 μM, or 1 μM for 24-hour treatment) or just pyocyanin (100 μM or 50 μM for 16-hour treatment). ALI samples were treated baso-laterally only. After exposure, cells were lysed with TRIzol reagent (ThermoFisher Scientific) and RNA extraction was carried out using the DirectZol RNA Kit (Thermo Fisher Scientific; Waltham, MA) following the manufacturer's instructions. Complementary DNA (cDNA) was synthesized using the High-Capacity cDNA Reverse Transcriptase Kit (ThermoFisher Scientific) in a Thermo-Cycler (Techne TC-512, Keison Products). RNA expression was quantified using TaqMan qPCR probes (QuantStudio 5 Real-Time PCR System, ThermoFisher Scientific). All expression levels were quantified using the $2^{-\Delta\Delta CT}$ method and were compared to either UBC (RPMI2650), or Rpl13a (Submerged HNEC)

Proteasomal degradation measurement

RPMI2650, NCI-H520, or submerged HNEC were transfected as described above but with UbG76V-eGFP-V5His_20aa (Addgene Plasmid #23970;[10]) hereafter referred to as Ub^{G76V}-GFP_{V5His}. Cells were treated with either control (media) or 150 μM pyocyanin for 4 hours. 12 random images in each cell type per treatment were captured on an Olympus IX-83 microscope (20x 0.75 NA objective), FITC filters (Chroma Technologies), Orca Flash 4.0 sCMOS camera (Hamamatsu, Tokyo, Japan) and MetaMorph (Molecular Devices; Sunnyvale, CA USA).

***Pseudomonas aeruginosa* culturing and pyocyanin extraction**

Pseudomonas aeruginosa culture Boston 41501 (ATCC 27853) was grown in lysogeny broth (LB) while *P. aeruginosa* cultures PAO1 (ATCC BAA-47), P11006, and PGO2338 (ATCC BAA-2110) were grown in King's A medium in Bio-Reaction tubes (CellTreat; Ayer, Massachusetts, USA) at 250 rpm between 30-37°C for 4 days and extracted as described.[11] Extracted pyocyanin was measured via absorbance at 520 nm (Tecan; Männedorf, Switzerland), and concentration (μg/mL) calculated as described.[12]

Statistical Analysis

All data were analyzed for statistical significance using unpaired, two-tailed t-tests (2 comparisons) or one-way ANOVA (more than 2 comparisons) in GraphPad PRISM version 10 (San Diego, CA, USA) unless specified otherwise. Dunnett's, Bonferroni, or Tukey's post tests were used for one-way ANOVA when appropriate as indicated in figure legends. In each of the figures, $p < 0.05$ (*), $p < 0.01$ (**), $p < 0.001$ (***), and no statistical significance (N.S or unmarked). All data points represent the mean ± SEM of ≥3 in independent experiments. When primary HNECs were used, all data were repeated in ≥3 separate patients unless specified otherwise.

Supplemental Table

Table S1. TaqMan assays used for RNA quantification

Name of Gene	Assay ID
Activating transcription factor 6 (*ATF6*)	Hs00232586_m1
BCL2 associated X (*BAX*)	Hs00180269_m1
BCL2	Hs04986394_s1
Caspase 3 (*CASP3*)	Hs00234387_m1
Caspase 7 (*CASP7*)	Hs00169152_m1
EDEM1	Hs00976004_m1
EDEM2	Hs01076556_m1
EDEM3	Hs00981767_m1
Heme oxygenase 1 (*HMOX1*)	Hs01110250_m1
Interleukin-1β (*IL1B*)	Hs01555410_m1
Interleukin-6 (*IL6*)	Hs00174131_m1
Interleukin-8 (*CXCL8*)	Hs00174103_m1
Interleukin-33 (*IL33*)	Hs04931857_m1
NAD(P)H dehydrogenase quinone 1 (*NQO1*)	Hs01045995_m1
Superoxide Dismutase II (*SOD2*)	Hs00167309_m1
Tumor necrosis factor (*TNF*)	Hs00174128_m1
Thymic stromal lymphopoietin (*TSLP*)	Hs00263639_m1
Ribosomal protein L13a (*RPL13A*)	Hs04194366_g1
Ubiquitin C (*UBC*)	Hs00824723_m1

Supplemental Figures

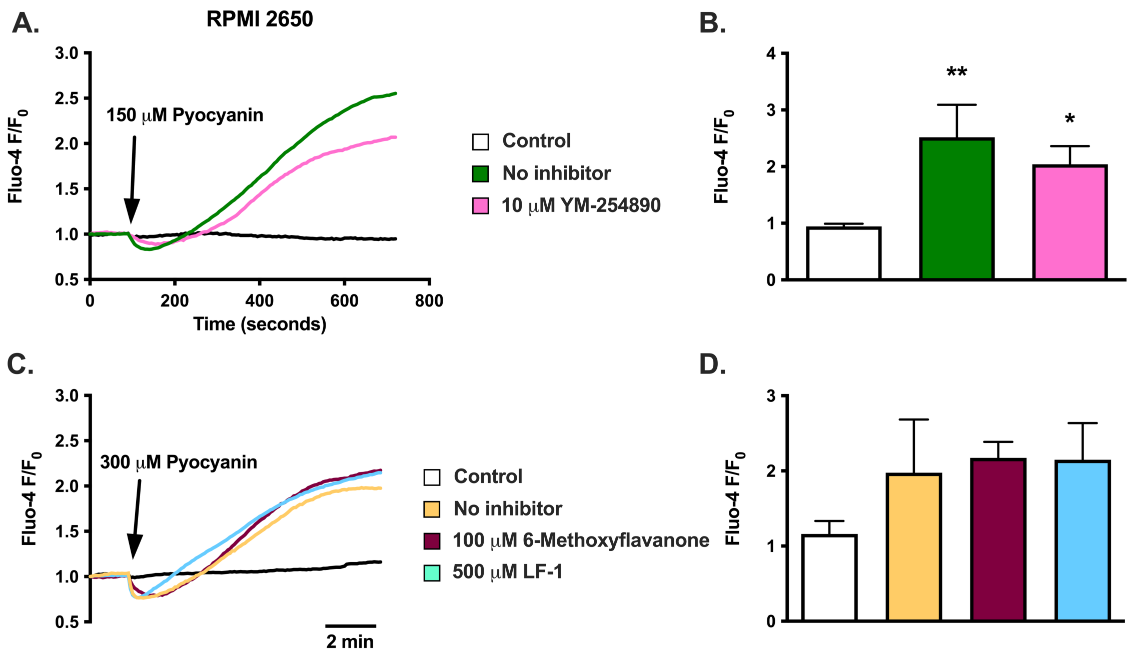

Figure S1. Pyocyanin does not activate cytosolic calcium responses via Gαq or T2R14 signaling. *A*). Average traces (n ≥ 3 independent experiments) of Fluo-4 intracellular calcium responses in RPMI2650 that were pre-treated for 1 hour with YM-254890, a Gαq inhibitor. 150 μM pyocyanin induced calcium responses in both YM-254890 treated and non-treated cells, while control (HBSS) had no effect in either treatment group. *B*). Intracellular Ca^{2+} (mean ± SEM) 10 minutes post treatment in RPMI2650. *C*). Average traces (n ≥ 3 independent experiments) of Fluo-4 intracellular calcium responses in RPMI2650 that were pre-treated for 1 hour with 6-methoxyflavanone (6-MF) or LF-1, both T2R14 inhibitors. 300 μM pyocyanin induced calcium responses in 6-MF and LF-1 treated and non-treated cells, while control (HBSS) had no effect in any treatment group *D*). Intracellular Ca^{2+} (mean ± SEM) 10 minutes post treatment in RPMI2650. Significance in bar graphs determined by one-way ANOVA with Dunnett's posttest; *p<0.05, **p<0.01; N.S. not significantly different.

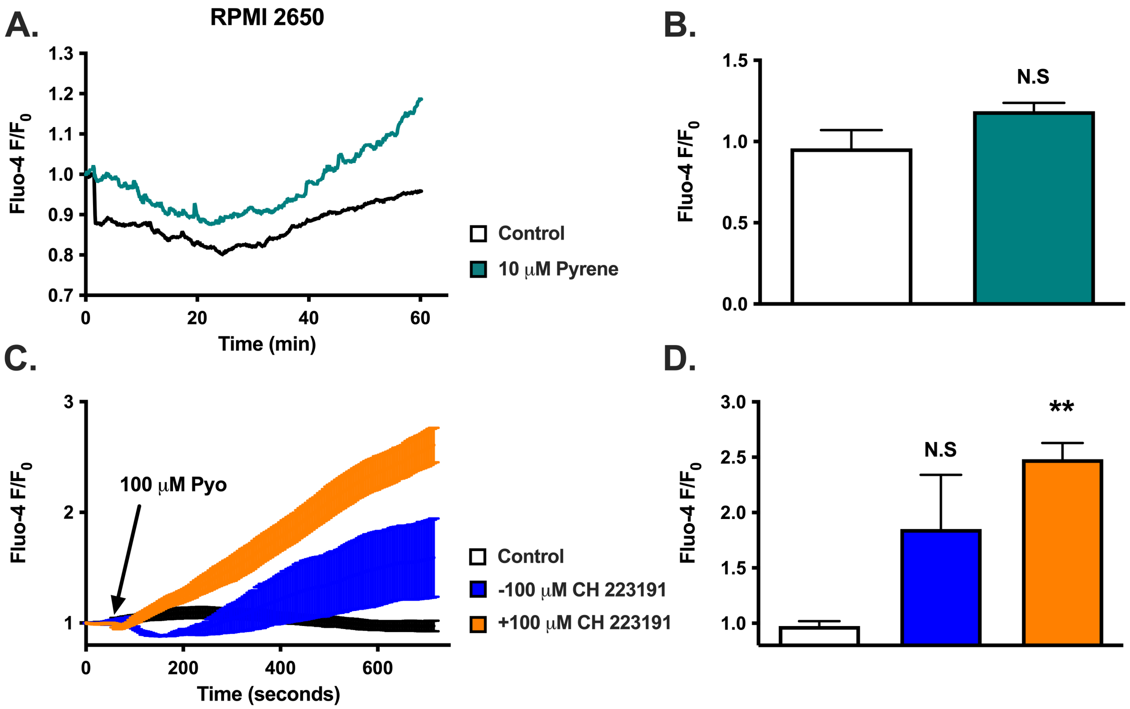

Figure S2. Pyocyanin does not activate cytosolic calcium responses via the acyl hydrocarbon receptor (AHR). *A*). Average traces (n ≥ 2 independent experiments) of Fluo-4 intracellular calcium responses in RPMI2650 that were treated with 10 µM pyrene, a known AHR agonist, or control (HBSS). *B*). Intracellular Ca^{2+} (mean ± SEM) 1-hour post treatment in RPMI2650. 10 µM pyrene was not significant compared to control. *C*). Average traces (n ≥ 3 independent experiments), with error bars, of Fluo-4 intracellular calcium responses in RPMI2650 that were pre-treated for 1 hour with CH 223191, an AHR specific inhibitor. 100 µM pyocyanin induced calcium responses in CH223191 treated and non-treated cells, while control (HBSS) had no effect in any treatment group *D*). Intracellular Ca^{2+} (mean ± SEM) 10 minutes post treatment in RPMI2650. Cells pretreated with CH 223191 seemed to have increased calcium responses compared to non-pretreated cells. *A*). Average traces (n ≥ 3 independent experiments), with error bars, of tetramethylrhodamine ethyl ester (TMRE) dye fluorescence over time and *B*). mean ± SEM after 16 minutes in RPMI2650 after treatment with 10 µM pyrene and control (HBSS). Significance in bar graphs determined by unpaired t test (*B*) one-way ANOVA with Dunnett's posttest (*D*); **p<0.01; N.S. not significantly different.

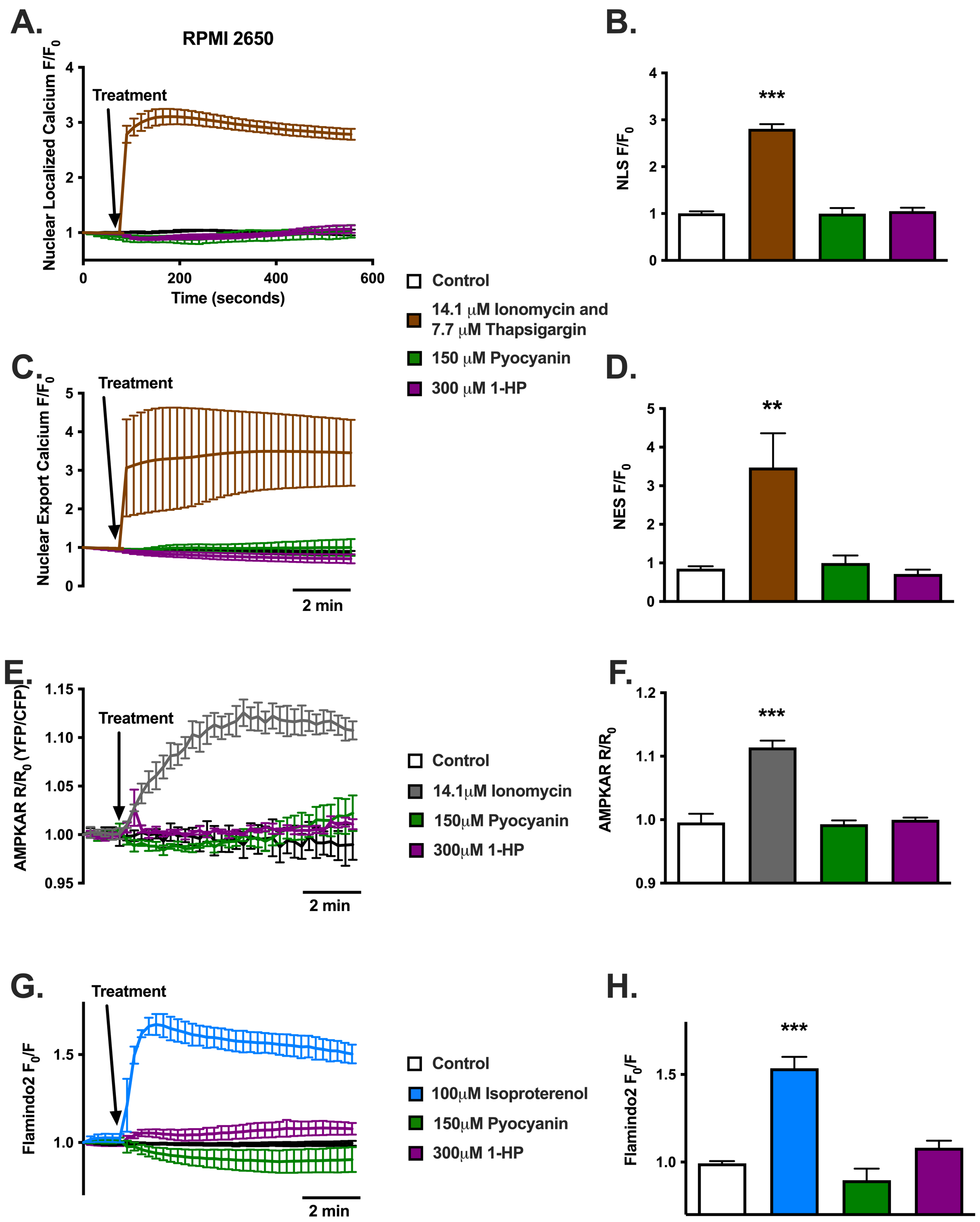

Figure S3. Pyocyanin and 1-hydroxyphenazine do not acutely activate nuclear calcium, AMP-activated protein kinase, or cyclic AMP in RPMI2650. *A* and *C*). Average traces (n ≥ 3 independent experiments), with error bars, of nuclear calcium responses in RPMI2650 transfected with *A*). NLS-R-Geco (nuclear localization signal) or **C).** NES-R-Geco (nuclear export signal). The combination of ionomycin and thapsigargin induced both localization and export signals, but pyocyanin and 1-hydroxyphenazine did not activate any response over a period of 8 minutes. *B*). Nuclear localization (NLS) or *D*). nuclear export signal (NES) (mean ± SEM) 7 minutes post treatment in RPMI2650.

Ionomycin and thapsigargin increased NLS and NES Ca^{2+} (Ca^{2+}_{nuc}) while pyocyanin or 1-HP did not. *E*). Average traces (n ≥ 3 independent experiments), with error bars, of AMP-activated kinase (AMPK) activity in RPMI2650 transfected with AMPKAR. Ionomycin increased AMPK activity while neither pyocyanin nor 1-hydroxyphenazine showed any increase. *F*). AMPKAR (mean ± SEM) 3.5 minutes post treatment in RPMI2650. *G*). Average traces (n ≥ 2 independent experiments), with error bars, of cyclic AMP (cAMP) activity in RPMI2650 transfected with Flamindo2. Isoproterenol increased cAMP activity, but neither pyocyanin nor 1-hydroxyphenazine changed cAMP activity. *H*). cAMP (mean ± SEM) 7 minutes post treatment in RPMI2650. Only isoproterenol increased cAMP activity while both pyocyanin and 1-HP did not. Significance in bar graphs determined by one-way ANOVA with Dunnett's posttest; **p<0.01, ***p<0.001; N.S. not significantly different.

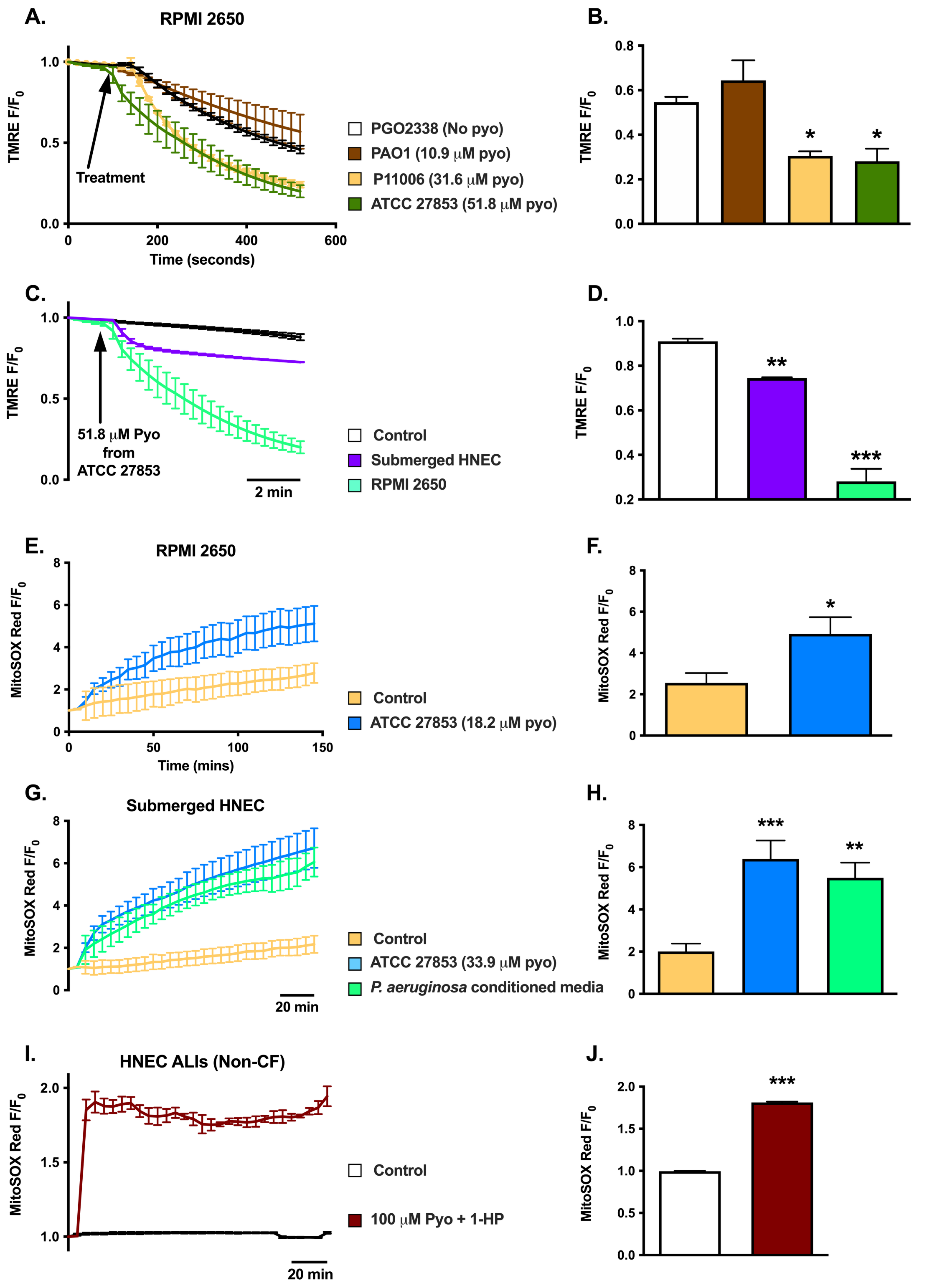

Figure S4. Extracted pyocyanin from several strains of *Pseudomonas aeruginosa* induces mitochondrial membrane depolarization and superoxide production in RPMI2650 and submerged HNECs. *A*). Average traces ($n \geq 3$ independent experiments), with error bars, of TMRE dye fluorescence over time and *B*). mean ± SEM

after 5 minutes in RPMI2650 treated with PGO2338 (0 μM pyocyanin), PAO1 (10.9 μM pyocyanin), P11006 (31.6 μM pyocyanin), or ATCC 27853 (51.8 μM pyocyanin). As expected, treatments from “higher” concentrations of pyocyanin resulted in larger decreases in mitochondrial membrane potential which were from P11006 and ATCC 27853. Interestingly, PAO1, which had extracted pyocyanin albeit at a low concentration, had similar decreases in mitochondrial membrane potential as PGO2338, which produced no pyocyanin. *C*). Average traces (n ≥ 3 independent experiments), with error bars, of TMRE dye fluorescence over time and *D*). mean ± SEM after 5 minutes in RPMI2650 or submerged HNEC treated with ATCC 27853 (51.8 μM pyocyanin) or vehicle control (LB). RPMI2650 had much larger decreases in mitochondrial membrane potential compared to submerged HNEC, which may indicate non-cancer cells are more resistant to the effects of extracted pyocyanin. Submerged HNEC was n = 1 individual, n ≥ 3 wells. *E-H*). Average traces (mean ± SEM; *E* or *G*) of MitoSOX Red fluorescence over time and bar graph (mean ± SEM; *F* or *H*) of fluorescence after 2 hours in *F*). RPMI2650 or *H*). Submerged HNEC after treatment with HBSS (LB), pyocyanin extracted from ATCC 27853 (18.2 μM in RPMI2650 or 33.8 μM in Submerged HNEC), or *Pseudomonas aeruginosa* conditioned media (Submerged HNEC) (n ≥ 3 independent experiments) *I-J*). Average traces (mean ± SEM; *E*) of MitoSOX Red fluorescence over time and bar graph (mean ± SEM; *F*) of fluorescence after 2 hours in HNEC ALI (non-CF) after treatment with HBSS (control) 100 μM pyocyanin + 1-HP (n ≥ 3 independent experiments). Significance in bar graphs determined by one-way ANOVA with Dunnett’s posttest; *p<0.05, **p<0.01, ***p<0.001; N.S. not significantly different.

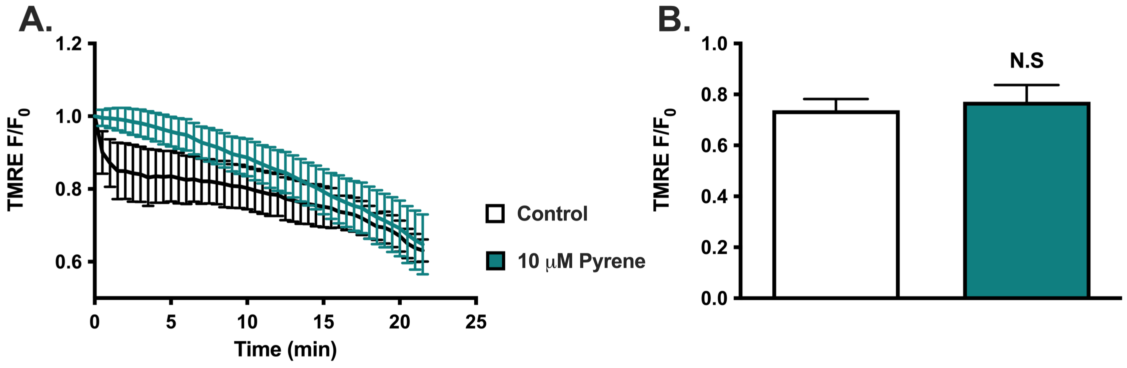

Figure S5. Pyocyanin does not decrease mitochondrial membrane potential via the AHR. *A*). Average traces (n ≥ 3 independent experiments), with error bars, of tetramethylrhodamine ethyl ester (TMRE) dye fluorescence over time and *B*). mean ± SEM after 16 minutes in RPMI2650 after treatment with 10 μM pyrene or control (HBSS). Significance in bar graphs determined by unpaired t test; N.S. not significantly different.

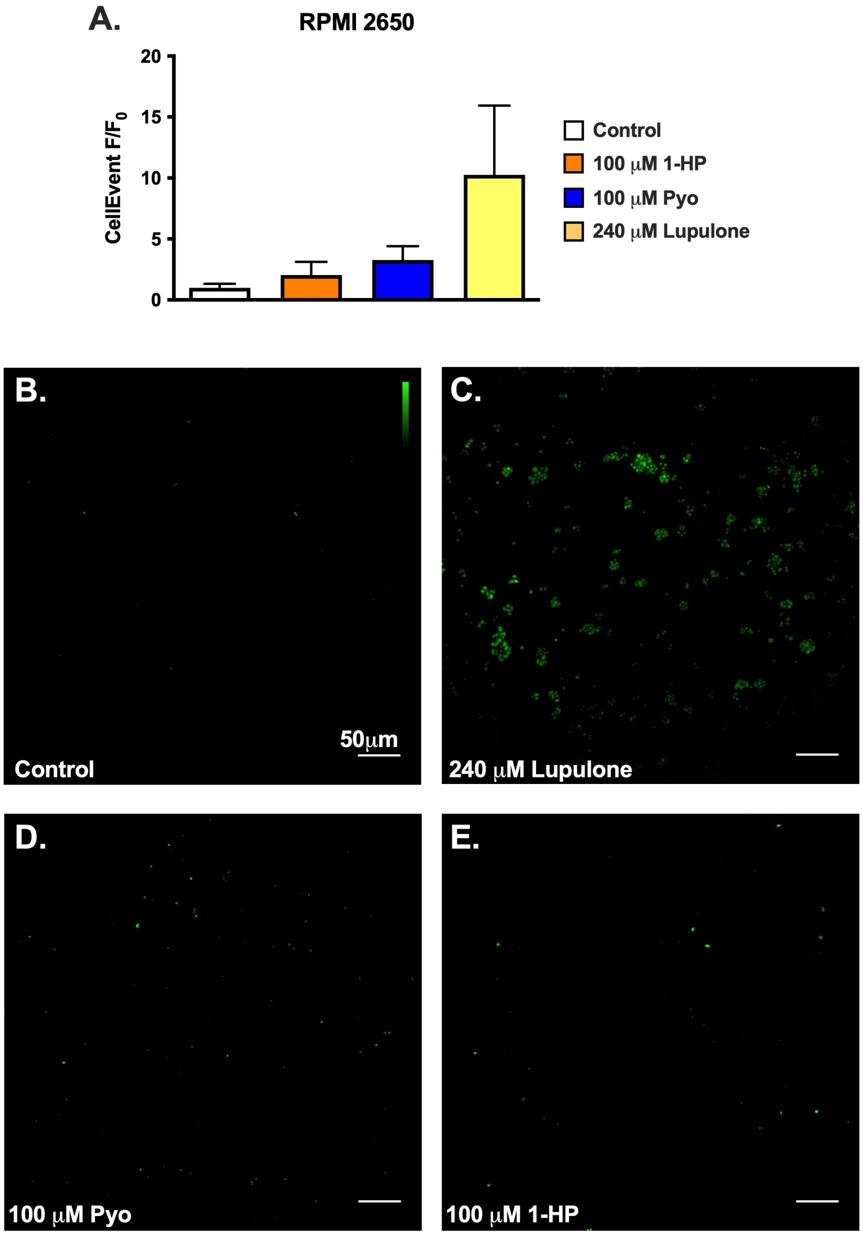

Figure S6. Pyocyanin and 1-hydroxyphenazine do not induce apoptosis through caspase 3 and 7 cleavage. *A*). CellEvent Caspase 3/7 (mean ± SEM) 21 hours post treatment in RPMI2650 (n = 3 independent experiments). Lupulone increased CellEvent F/F_0, although not significantly, while pyocyanin and 1-hydroxyphenazine did not seem to increase caspase 3/7 cleavage. Representative images at 21 hours post treatment in RPMI2650 for *B*). control, *C*). 240 μM lupulone, *D*). 100 μM pyocyanin, and *E*). 100 μM 1-hydroxypehanzine. Images were captured on a 20x objective and FITC filter. All scale bars are 50 μm. Significance in bar graphs determined by one-way ANOVA with Dunnett's posttest; N.S. not significantly different.

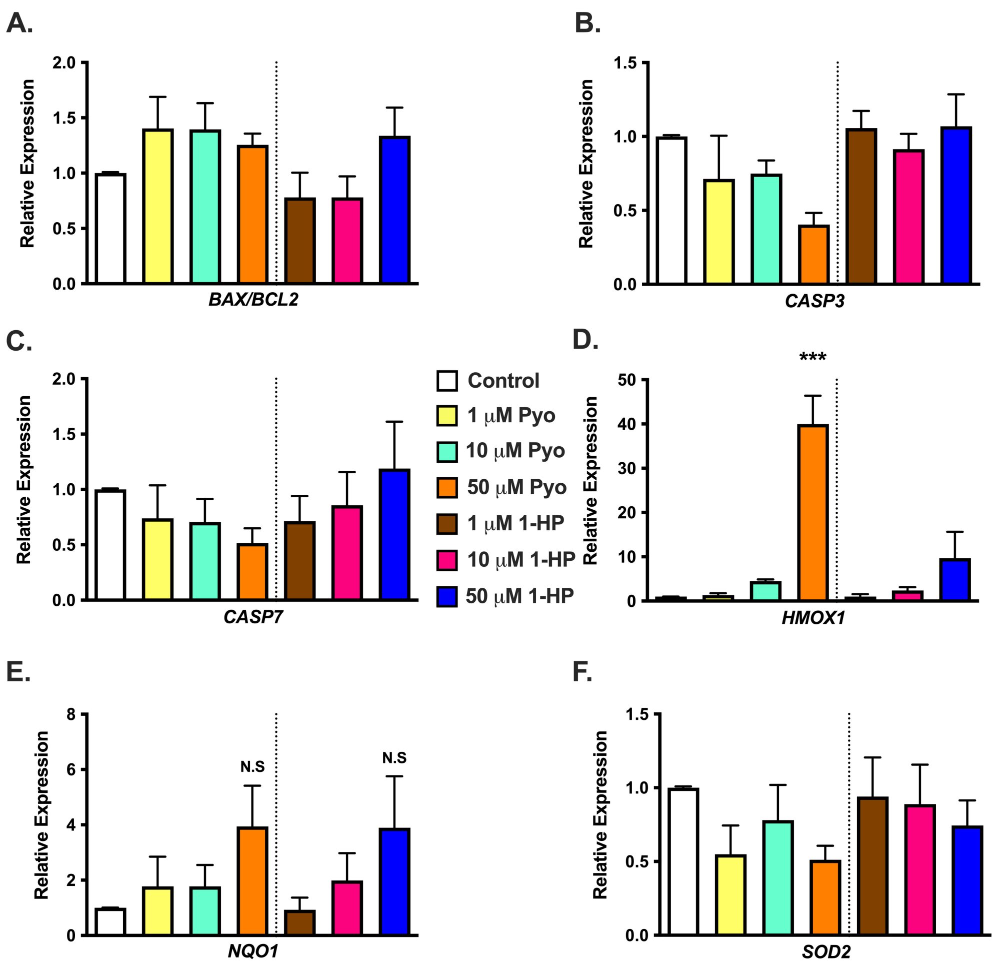

Figure S7. Pro-apoptosis and mitochondrial stress markers expression in RPMI2650 after 24-hour treatment with pyocyanin and 1-hydroxyphenazine. *A*). *BAX*/*BCL2*, *B*). *CASP3*, *C*). *CASP7*, *D*). *HMOX1*, *E*). *NQO1*, and *F*). *SOD2* expression relative to *UBC* in RPMI2650 after 24-hour exposure to various concentrations of pyocyanin or 1-hydroxyphenazine (n = 3 independent experiments). Pyocyanin induced increases in *BAX*/*BCL2* dose-dependently, although not statistically significant, while only the highest concentration of 1-hydroxyphenazine seemed to increase this ratio. Full length caspase 3 (*CASP3)* and 7 (*CASP7)* increases after exposure to 1-hydroxyphenazine, yet decreases after exposure from pyocyanin. *HMOX1* expression was increased significantly in the highest concentration of pyocyanin. NAD(P)H quinone dehydrogenase 1 (*NQO1*) increased dose-dependently after exposure to either pyocyanin or 1-hydroxyphenazine. Superoxide dismutase (*SOD2*) levels were largely unchanged, with only slight decreases seen in some concentrations of pyocyanin. The *BAX*/*BCL2* ratio is an indicator of apoptosis which is regulated through EGFR pathway. Upregulation of *BAX* is pro-apoptotic while *BCL2* is anti-apoptotic and an increase in the ratio between them indicates upregulation of *BAX* and downregulation of *BCL2*. Caspase 3 (*CASP3)* and 7 (*CASP7*) are cleaved during apoptosis, so decreases in full length expression would indicate active caspase proteins. Heme oxygenase 1 (*HMOX1)* expression, which codes for the HO-1 enzyme and is regulated by *Nrf2*, is upregulated during oxidative stress[13]. Significance in bar graphs determined by one-way ANOVA with Dunnett's posttest; ***$p<0.001$; N.S. not significantly different.

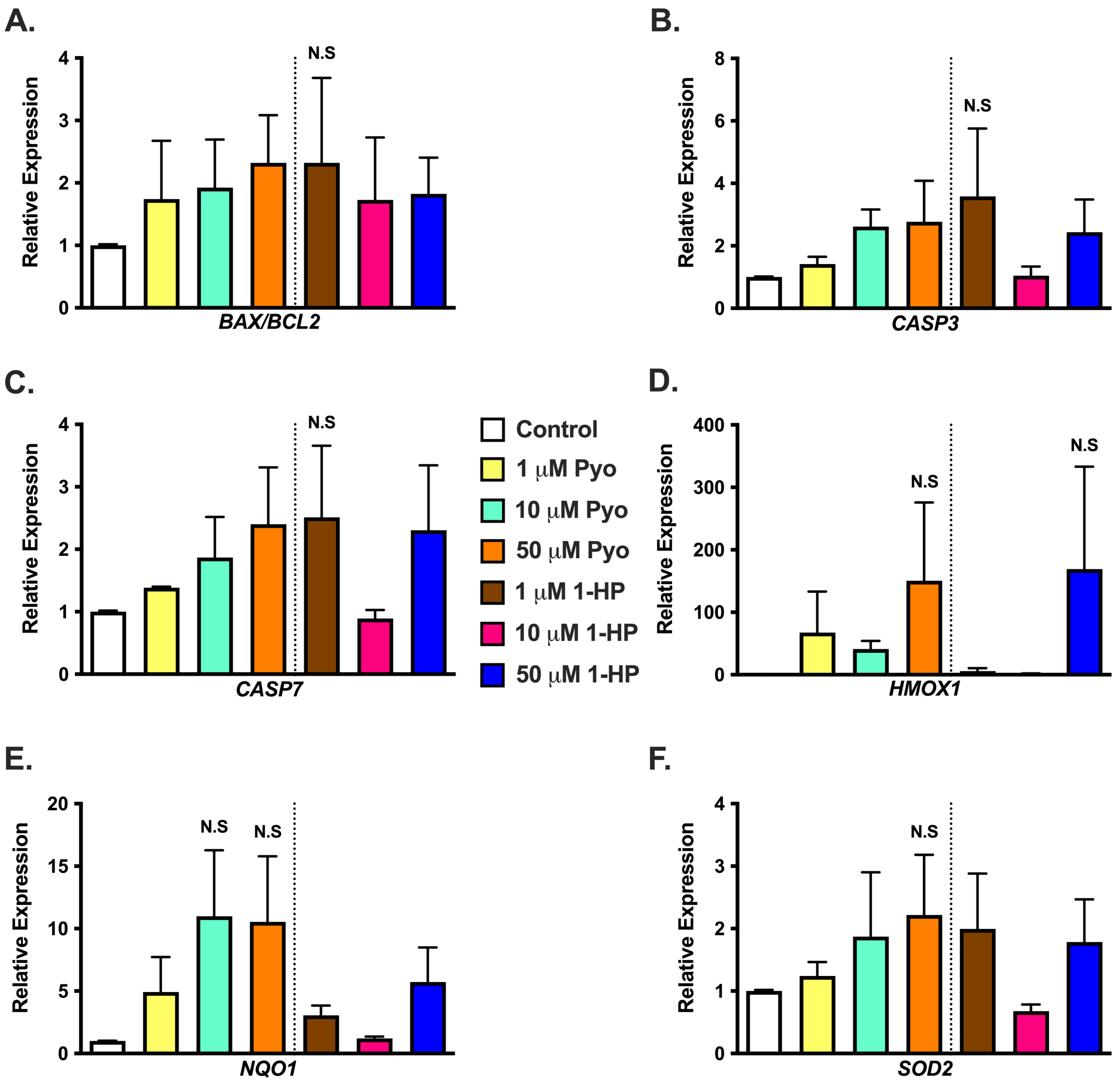

Figure S8. Pro-apoptosis and mitochondrial stress markers expression in submerged HNEC after 24-hour treatment with pyocyanin and 1-hydroxyphenazine. *A*). *BAX*/*BCL2*, *B*). *CASP*3, *C*). *CASP7*, *D*). *HMOX1*, *E*). *NQO1*, and *F*). *SOD2* expression relative to *Rpl13a* in submerged HNEC after 24-hour exposure to various concentrations of pyocyanin (Pyo) or 1-hydroxyphenazine (1-HP) (n = 1 independent experiments from using cells from 3 different patients). Pyocyanin induced increases in *BAX*/*BCL2* in dose-dependent manner, although not statistically significant, while 1-HP seemed to be reverse dose-dependent. Caspase 3 (*CASP3*) and 7 (*CASP7*) expression was increased dose-dependently from pyocyanin exposure which differs from results seen in RPMI2650 (Figure S4*A-B*). *HMOX1* expression was increased, although not significantly, in the highest concentration of 1-HP, and most pyocyanin concentrations. *NQO1* expression was increased from pyocyanin exposure, which was not seen to the same degree from 1-HP exposure. Superoxide dismutase II (*SOD2*) expression was increased dose dependently, and from certain concentrations of 1-HP, which differed from results seen in RPMI2650 (Figure S4*F*). Significance in bar graphs determined by one-way ANOVA with Dunnett's posttest; ***$p<0.001$; N.S. not significantly different.

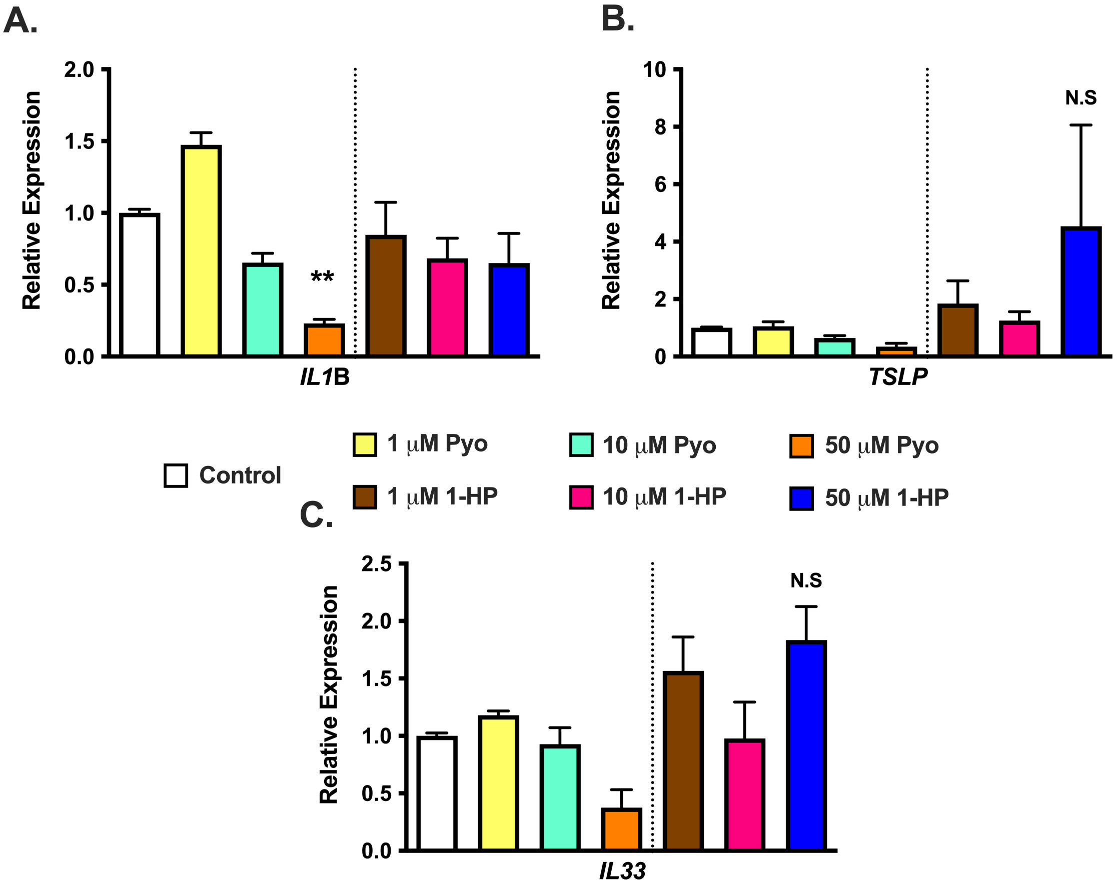

Figure S9. Cytokine expression in RPMI2650 after 24-hour treatment with pyocyanin or 1-hydroxyphenazine. *A*). *IL1B*, *B*). *TSLP*, and *C*). *IL33* expression relative to *UBC* in RPMI2650 after 24-hour exposure to various concentrations of pyocyanin or 1-hydroxyphenazine (n = 3 independent experiments). The highest concentration of pyocyanin statistically significantly decreased *IL1B* expression, while 1-hydroxyphenazine exposure did not change expression. *TSLP* and *IL33* expression increased, although not statistically significantly, after exposure to 1-hydroxyphenazine, yet decreased in both cases dose-dependently. Significance in bar graphs determined by one-way ANOVA with Dunnett's posttest; N.S. not significantly different.

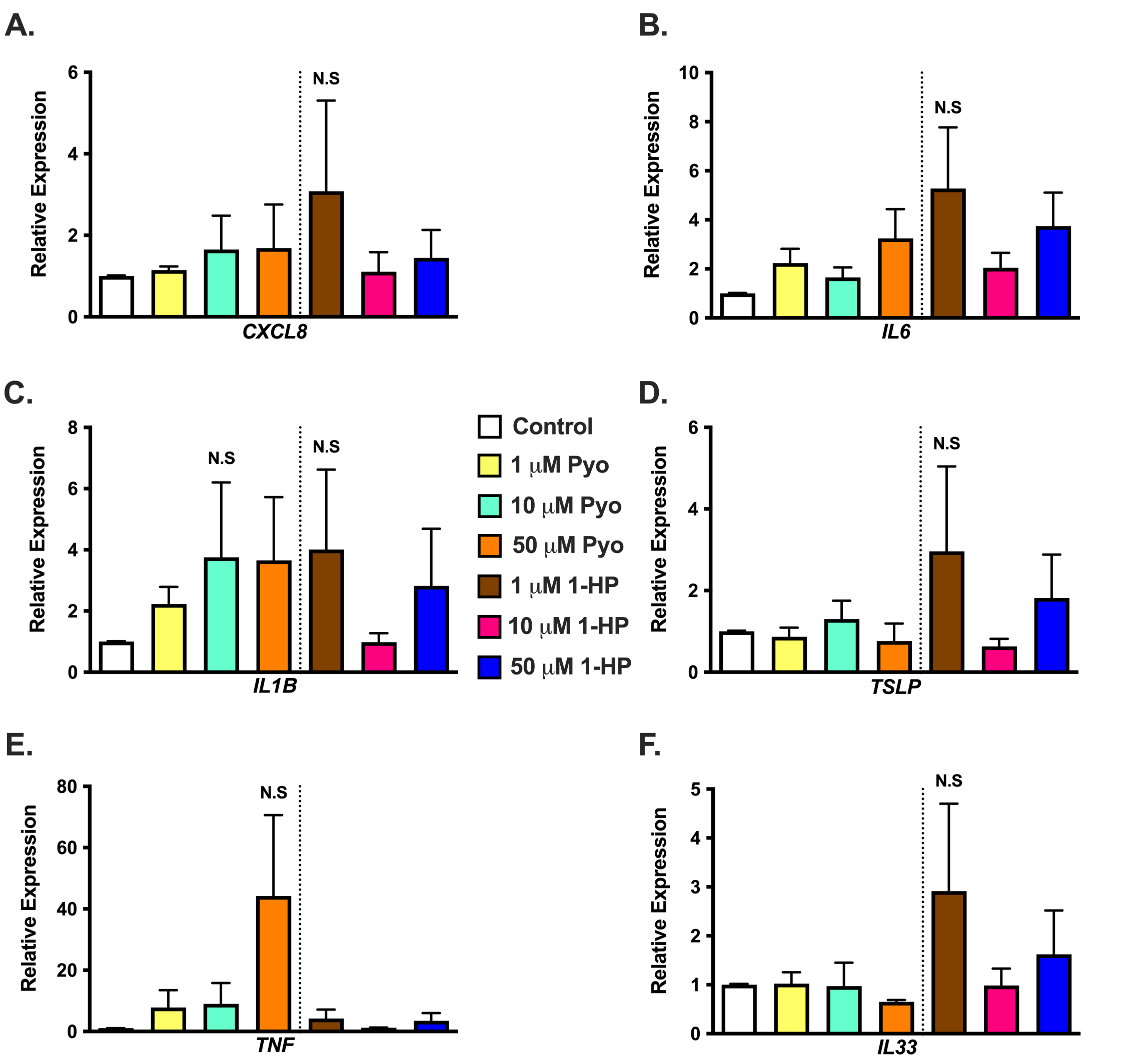

Figure S10. Cytokine expression in Submerged HNEC after 24-hour treatment with pyocyanin or 1-hydroxyphenazine. *A*). *CXCL8*, *B*). *IL6*, *C*). *IL1B*, *D*). *TSLP*, *E*). *TNF*, and *F*). *IL33* expression relative to *Rpl13a* in submerged HNEC after 24-hour exposure to various concentrations of pyocyanin or 1-hydroxyphenazine (n = 1 independent experiments from using cells from 3 different patients). *CXCL8* expression was slightly increased in higher concentration of pyocyanin, but was highest in the lowest concentration of 1-hydroxyphenazine, although this was not statistically significant. *Il6* and *IL1B* levels were elevated in most concentrations of pyocyanin and 1-hydroxyphenazine. Only the highest concentration of pyocyanin increased *TNF*, although this was not significant. *TSLP* and *IL33* expression were largely unaffected by pyocyanin, but some concentrations of 1-hydroxyphenazine increased expression. Significance in bar graphs determined by one-way ANOVA with Dunnett's posttest; N.S. not significantly different.

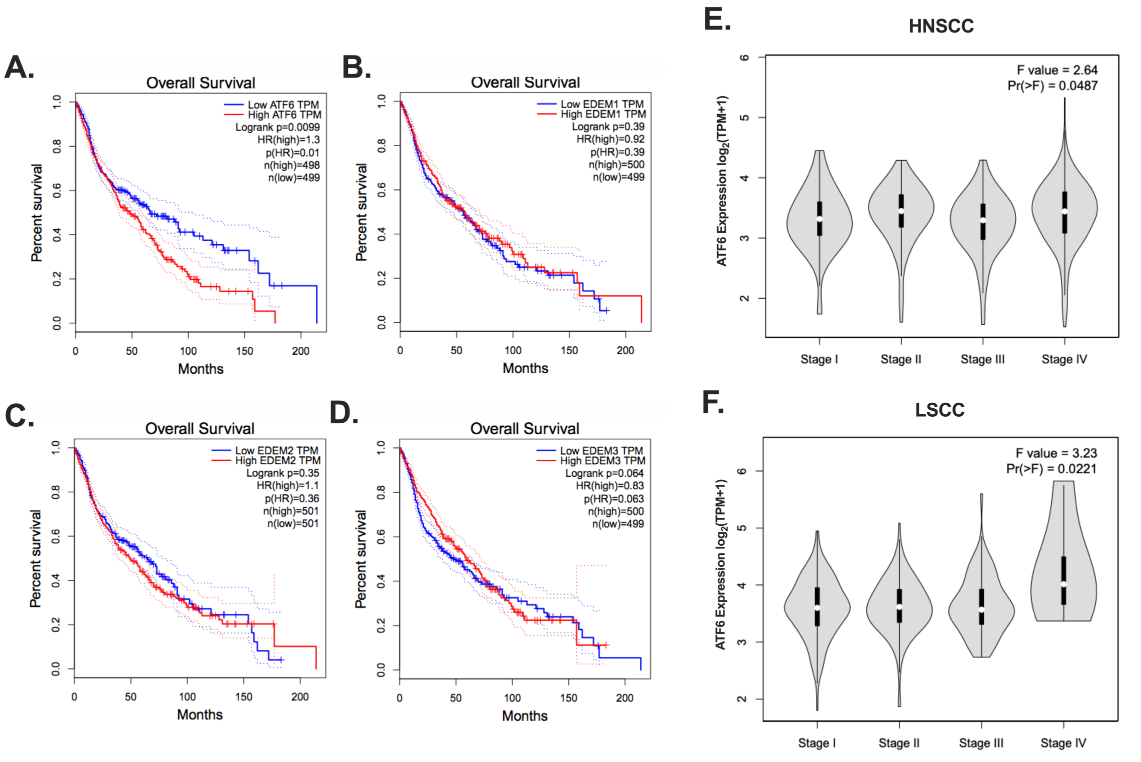

Figure S11. Stage plots of *ATF6* expression and survival curves of UPR^{ER} and ERAD genes in head and neck squamous cell carcinomas (HNSCCs) and lung squamous cell carcinomas (LSCCs). *A-B*). Pathological stage plots of *ATF6* expression (Log_2(TPM+1)) over stages I-IV in *A*). HNSCCs or *B*). LSCCs. *C-F*). Survival curves of *ATF6* (*C*), *EDEM1* (*D*), *EDEM2* (*E*), and *EDEM3* (*F*) in HNSCCs and LSCCs over <16 years. Data and analysis from all figures were completed on GEPIA2.[14]

Supplemental References

1. Wu J, Prole DL, Shen Y, et al. Red fluorescent genetically encoded Ca2+ indicators for use in mitochondria and endoplasmic reticulum. Biochem J 2014; 464:13-22.
2. Palmer AE, Jin C, Reed JC, et al. Bcl-2-mediated alterations in endoplasmic reticulum Ca2+ analyzed with an improved genetically encoded fluorescent sensor. Proc Natl Acad Sci U S A 2004; 101:17404-17409.
3. Mehta S, Zhang Y, Roth RH, et al. Single-fluorophore biosensors for sensitive and multiplexed detection of signalling activities. Nat Cell Biol 2018; 20:1215-1225.
4. Ribeiro CM, Paradiso AM, Livraghi A, et al. The mitochondrial barriers segregate agonist-induced calcium-dependent functions in human airway epithelia. J Gen Physiol 2003; 122:377-387.
5. Kouakou YI, Thompson JC, Tan LH, et al. Hops bitter beta-acids have antibacterial effects against sinonasal Staphylococcus aureus but also induce sinonasal cilia and mitochondrial dysfunction. Int Forum Allergy Rhinol 2024.
6. Zhao Y, Araki S, Wu J, et al. An expanded palette of genetically encoded Ca(2)(+) indicators. Science 2011; 333:1888-1891.
7. Tsou P, Zheng B, Hsu CH, et al. A fluorescent reporter of AMPK activity and cellular energy stress. Cell Metab 2011; 13:476-486.
8. Odaka H, Arai S, Inoue T, et al. Genetically-encoded yellow fluorescent cAMP indicator with an expanded dynamic range for dual-color imaging. PLoS One 2014; 9:e100252.
9. Sisson JH, Stoner JA, Ammons BA, et al. All-digital image capture and whole-field analysis of ciliary beat frequency. J Microsc 2003; 211:103-111.
10. Beskow A, Grimberg KB, Bott LC, et al. A conserved unfoldase activity for the p97 AAA-ATPase in proteasomal degradation. J Mol Biol 2009; 394:732-746.
11. Shouman H, Said HS, Kenawy HI, et al. Molecular and biological characterization of pyocyanin from clinical and environmental Pseudomonas aeruginosa. Microb Cell Fact 2023; 22:166.
12. Essar DW, Eberly L, Hadero A, et al. Identification and characterization of genes for a second anthranilate synthase in Pseudomonas aeruginosa: interchangeability of the two anthranilate synthases and evolutionary implications. J Bacteriol 1990; 172:884-900.
13. Dunn LL, Kong SMY, Tumanov S, et al. Hmox1 (Heme Oxygenase-1) Protects Against Ischemia-Mediated Injury via Stabilization of HIF-1alpha (Hypoxia-Inducible Factor-1alpha). Arterioscler Thromb Vasc Biol 2021; 41:317-330.
14. Tang Z, Kang B, Li C, et al. GEPIA2: an enhanced web server for large-scale expression profiling and interactive analysis. Nucleic Acids Res 2019; 47:W556-W560.
